# The molecular basis of socially mediated phenotypic plasticity in a eusocial paper wasp

**DOI:** 10.1038/s41467-021-21095-6

**Published:** 2021-02-03

**Authors:** Benjamin A. Taylor, Alessandro Cini, Christopher D. R. Wyatt, Max Reuter, Seirian Sumner

**Affiliations:** 1 Centre for Biodiversity & Environment Research, University College London, London, UK; 2 Department of Genetics, Evolution & Environment, University College London, London, UK; 3 Dipartimento di Biologia, Università degli Studi di Firenze, Sesto Fiorentino, Italy; 4 Centre for Life’s Origins and Evolution, University College London, London, UK

**Keywords:** Bioinformatics, Machine learning, Animal behaviour, Entomology

## Abstract

Phenotypic plasticity, the ability to produce multiple phenotypes from a single genotype, represents an excellent model with which to examine the relationship between gene expression and phenotypes. Analyses of the molecular foundations of phenotypic plasticity are challenging, however, especially in the case of complex social phenotypes. Here we apply a machine learning approach to tackle this challenge by analyzing individual-level gene expression profiles of *Polistes dominula* paper wasps following the loss of a queen. We find that caste-associated gene expression profiles respond strongly to queen loss, and that this change is partly explained by attributes such as age but occurs even in individuals that appear phenotypically unaffected. These results demonstrate that large changes in gene expression may occur in the absence of outwardly detectable phenotypic changes, resulting here in a socially mediated de-differentiation of individuals at the transcriptomic level but not at the levels of ovarian development or behavior.

## Abstract (web summary)

Connecting genotypes to complex social behaviour is challenging. Taylor et al. use machine learning to show a strong response of caste-associated gene expression to queen loss, wherein individual wasp’s expression profiles become intermediate between queen and worker states, even in the absence of behavioural changes.

## Introduction

The relationship between gene expression and external phenotype is complex and unresolved. Much research in behavioral and evolutionary ecology is based on the implicit assumption that phenotypic traits can be modeled as though they directly reflect gene expression patterns, and that evolutionary trajectories can therefore be studied while remaining agnostic with regard to the underlying molecular mechanisms^1–3^. This “phenotypic gambit” has proven a useful rule of thumb, permitting the establishment of a rich body of literature surrounding the evolution of complex traits, despite a lack of data relating to the genetic basis of these traits^4–6^. In the past decade, however, advances in the affordability of “omic” data and availability of powerful bioinformatic methods have greatly enhanced our ability to assess the assumptions made by the phenotypic gambit^2,7^. The time is right to disentangle the molecular foundations of complex phenotypic traits.

Phenotypic plasticity, the ability of an individual to effect phenotypic changes in response to external cues, is an ideal phenomenon with which to study the relationship between gene expression and phenotype because it involves the production of multiple phenotypes without gene sequence changes. Of particular, value are species in which adult individuals can be experimentally induced to transition between distinct, measurable phenotypes. By comparing the gene expression profiles of groups of individuals that differ in their phenotypes as a result of plasticity rather than as a result of genetic differences, it is possible to isolate phenotypic effects of gene expression. Using this approach, significant progress has been made in unraveling the molecular underpinnings of sequential sex changes in hermaphroditic fish^8–10^, the distinct gregarious social phenotype of desert locusts^11,12^, and the reproductive castes of social insects^13–17^. Such studies typically rely on comparisons between groups of individuals with well-differentiated phenotypes, however. As a result, little is known about more subtle effects during the transition from one morph to the other, and the relationship between expression patterns and phenotypic traits at the individual level.

The reproductive castes found in the colonies of social insects provide excellent model systems for determining the extent to which fine-scale changes in phenotype are reflected at the molecular level. With a few exceptions^18^, the distinct queen and worker phenotypes found in such colonies are plastically determined either during development or in adulthood. Workers in some species can be experimentally induced to transition to a reproductive role in response to the removal of a colony’s queen^19,20^ or as a result of exposure to varying levels of brood^15,21^, allowing changes in the behavioral, physiological, and molecular traits that define caste identity to be tracked. An additional benefit—and challenge—of studying social insect colonies is that they involve complex social structures. Such interactions can be hard to study, but offer the opportunity to assess the effects of social interactions upon phenotypes and transcriptomes.

**Fig. 1:**
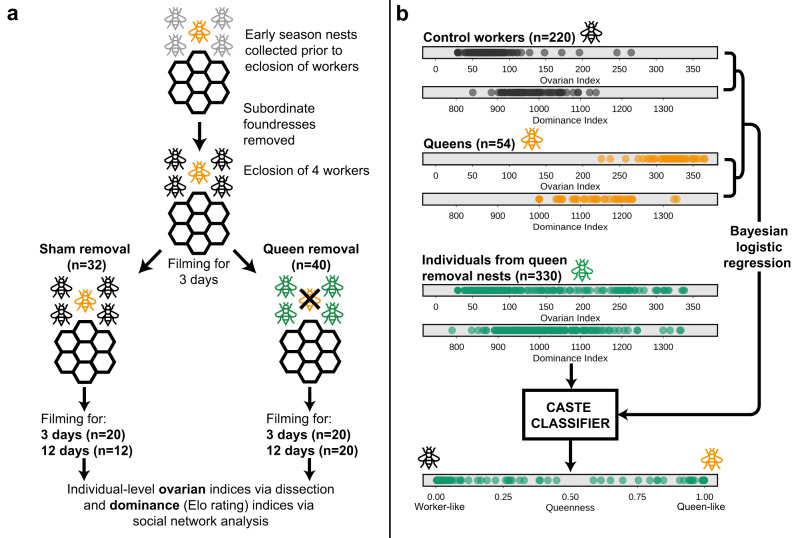
Summarized experimental design describing the generation of phenotypic data pertaining to individual-level responses to queen removal in *Polistes dominula*^29^ used in this study. **a** Early-season nests were transferred to the lab prior to the eclosion of workers and subordinate foundresses were removed. After at least 4 workers had eclosed, nests were filmed for 3 days and then assigned to either control or queen removal treatments. Following treatment (sham or queen removal) nests were filmed for a further 3 or 12 days and then all individuals were dissected to generate ovarian development indices following^57^. Footage of nests was used to generate dominance indices in the form of Elo ratings^59,60^. **b** Ovarian and dominance indices from queens (orange) and control workers (black) were used to produce a logistic regression model for caste classification (0 = worker, 1 = queen). Data from individuals on queenless post-removal nests (green) were then passed through this model to fit caste estimates and thereby identify individuals with high “queenness”, i.e., those that exhibited strongly queen-like phenotypes following queen removal and thus represented possible replacement queens. Of the individuals for which data are shown here, 27 queens, 12 workers from control nests, and 62 individuals from queen removal nests were subsequently sequenced to generate the data discussed in the present study. Images (wasps and nest combs) created by BAT.

The European paper wasp *Polistes dominula* (Christ 1791) is a model organism often used in studies of social insect behavior^22,23^ and, more recently, for analyses of caste gene expression^24–26^. In this species, removing the established queen from a single-foundress colony induces a queen succession process in which one (or very few) workers transition to a queen phenotype, with age playing a key role in predicting which individual will do so^27,28^: almost invariably, the new queen is one of the oldest individuals, and there is little conflict over succession^19,29^. In a recent paper^29^, we followed responses to queen removal in *P. dominula* on a fine scale by measuring individual-level behavioral and physiological traits and generating a univariate measure of caste identity (“queenness”) to describe individuals’ phenotypic profiles (Fig. 1). We found that phenotypic responses were limited to the subset of individuals that transitioned to the queen role, while other individuals exhibited little or no measurable change in measured traits. This system, in which individuals within a controlled environment vary strongly and predictably in their phenotypic response to a shared stimulus, affords an excellent opportunity to track the relationship between gene expression and phenotypic expression at the individual level.

Support vector classification is a powerful tool with which to transform complex patterns in multidimensional data into a continuous classification score, allowing the detection of subtle, widespread signals of differential expression between phenotypic states that are likely to be missed in conventional differential expression analyses. Support vector machines (SVMs) have become a key tool in the early identification of phenotypically indistinguishable cancer subtypes^30–32^ and their potential value has recently been demonstrated in animal behavior studies: Chakravarty et al.^33^, for example, recently showed that an SVM trained using accelerometer data can reliably classify the behaviors of wild Kalahari meerkats. Given their ability to detect subtle changes in patterns of high-dimensional data, SVMs should be ideally suited to quantify gene expression variation across the spectrum between differentiated worker and queen roles.

Here, we apply an SVM approach to analyze transcriptomes from the brains of 96 individuals for which we previously obtained fine-scale behavioral and ovarian data, including queens and workers from stable colonies and individuals from colonies that had their queens experimentally removed. Combining this approach with standard differential expression and gene co-expression analyses, we show that brain gene expression responses to queen removal in *P. dominula* include a colony-wide response that does not match that observed at the phenotypic level. Our results indicate that gene expression in *P. dominula* colonies reflects both a generalized response to queen loss that is seemingly independent of phenotype, and a phenotype-specific response that tracks individuals’ expression of plastic phenotypic changes. This study provides a comprehensive analysis of the ways that plastic phenotypes are reflected at the transcriptomic level; our results expose the complexity of the relationship between individual-level gene expression and individuals’ outward phenotypes.

## Results

### Support vector classification reveals consistent patterns of caste gene expression differentiation involving many genes

SVMs operate in a similar fashion to multivariate linear regression models, estimating the relationship between a response variable (here, known caste identities coded as worker = 0 and queen = 1) and one or more independent variables (here, expression profile) in a training data set. The model derived in this way is then applied to the query data set to derive predictions of the response. In contrast to standard linear models, SVM models project input data into a higher-dimensional space, thereby making it possible to fit a linear relationship to what would otherwise be non-linear data^34^. Using this approach allowed us to reduce the brain gene expression data we had generated down to a single dimension of predicted caste identity (the classifier variable), analogous to the Bayesian logistic regression model we used in Taylor et al.^29^ to condense ovarian and behavioral data into a unidimensional metric of phenotypic caste (“queenness”; Fig. 1).

We trained an SVM using whole-transcriptome data from 26 queens and 12 workers from stable, queenright colonies. A full model, based on all 10,734 genes annotated in our experiment, achieved a root mean squared validation error of 0.065 in threefold cross-validation, i.e., a model trained on a random subset of two-thirds of workers and queens classified the remaining third within 25.5% of their true values (0 and 1 for workers and queens, respectively). This error rate suggests reliable classification because it indicates that any given control worker was likely to receive a classification that was closer to that of all other workers than it was to the classification of any given queen, and vice versa.

**Fig. 2:**
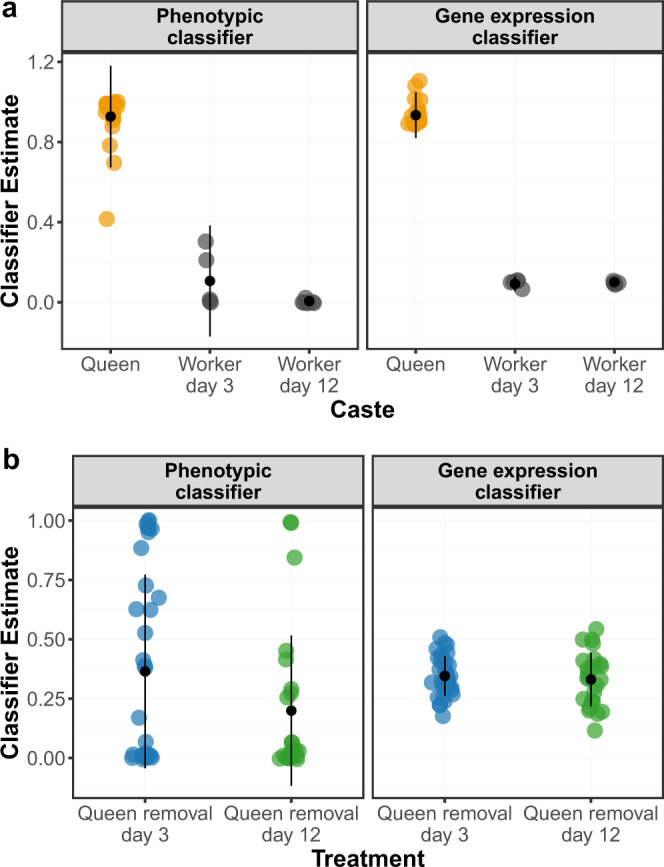
Phenotypic and gene expression classification of individuals from control and experimental colonies. Classifier estimates generated by a Bayesian logistic regression model using ovarian and dominance data (left) and an SVM model using 1992 caste-informative genes (right) for **a** queens (orange; *n* = 26 biologically independent samples) and workers (black; *n* = 12 biologically independent samples) from control colonies and **b** individuals from experimental colonies either 3 days (blue; *n* = 24 biologically independent samples) or 12 days (green; *n* = 34 biologically independent samples) following queen removal. Mean and standard deviation for each group are shown in black. Classifications are a measure of individuals’ phenotypic/gene expression similarity to queens and workers from control nests. 0 = complete similarity to control workers; 1 = complete similarity to control queens.

As many genes will not vary consistently in their expression between workers and queens, an SVM model fit to the entire transcriptome is likely to exhibit a significant degree of overfitting. In order to identify a minimal set of genes that were maximally predictive of caste identity, we applied a process of “feature selection” in which uninformative genes were progressively dropped until an optimal model containing only caste-informative genes was achieved (Supplementary Fig. S1; Supplementary Data File 1). The model obtained in this way contained 1992 genes with a root mean squared classification error of 0.021, a substantial improvement over that achieved for the model containing all genes (Supplementary Data File 2). This model classified queens and workers very consistently, with strong separation of queens from workers (Fig. 2A). Thirty-four gene ontology (GO) terms were significantly enriched among these 1992 genes, including a number of terms associated with translation such as rRNA processing, tRNA aminoacylation, and ribosomal large subunit biogenesis (Supplementary Fig. S2).

When we applied a more standard differential expression approach based on DESeq2 analysis and a 1.5-fold-change threshold to the same set of queens and workers, we identified just 81 differentially expressed genes (with no associated GO terms), a number that is typical for similar analyses in *Polistes*^25,35,36^. Of these genes, 77/81 (95%) were present in the larger SVM set (Supplementary Fig. S3; Supplementary Data File 3), suggesting that the SVM captures the information contained in the set of highly caste-differentiated genes identified using this standard method. Yet, an SVM trained using just these 81 differentially expressed genes exhibited a cross-validation error rate of 0.065, significantly higher than that of the optimized model (0.021) and no better than the original un-optimized model containing all expressed genes. Thus, the picture of caste differentiation provided by standard differential expression analysis appears to miss a great number of subtle differences in individuals’ gene expression profiles that contribute to caste differentiation.

The small set of differentially expressed genes that we identified using DESeq2 in the above analysis might conceivably reflect the conventional fold-change threshold that was applied as part of the approach. Indeed, when we omitted this cutoff, we found 2438 differentially expressed genes, on the same order of magnitude as that found via SVM feature selection. This set of genes overlapped significantly with those identified by feature selection (Jaccard index = 0.44; one-sided hypergeometric overlap *p* < 0.001). However, an SVM model trained using the differentially expressed genes exhibited a root mean squared error rate of 0.030, and thus still performed substantially less well than the model using the genes identified using feature selection (0.021).

### Colony-wide brain gene expression responses to queen removal

Following the loss of a queen from a *Polistes* colony, typically one or a few individuals undergo a phenotypic transition to become a replacement queen while the rest of the colony members remain workers^29,37,38^. To capture this transition at the transcriptional level, we analyzed individuals’ gene expression profiles at three days after queen removal, when queen replacement is ongoing (*n* = 24), and at 12 days after queen removal (*n* = 34), when succession is largely settled at the phenotypic level^19,29^. Individuals for sequencing were selected to cover a wide range of phenotypes, including those that remained entirely worker-like, those that had transitioned to highly queen-like phenotypes, and those with intermediate phenotypes at the time of sampling (Supplementary Fig. S4).

In order to assess changes in individuals’ caste-specific gene expression profiles following queen removal, we applied the optimized SVM model described above to the gene expression profiles of these 62 individuals. Doing so generated an SVM classification for each individual that describes the degree to which its gene expression corresponds to the worker state (classifier = 0) or queen state (classifier = 1) as reflected in the expression profiles of the control workers and queens on which the SVM was trained.

Analyzing shifts in SVM estimates of individual wasps then allowed us to assess the degree to which these concurred with the changes visible at the phenotypic level. Doing so, we found that the SVM estimates of individuals from post-removal nests were intermediate between those of queens and workers from control nests (Fig. 2B), a finding which concurs with the placement of these individuals according to principal component analysis (Supplementary Fig. S5). This result is surprising given that the majority of individuals on queen removal nests are phenotypically indistinguishable from workers on control nests in terms of ovarian development and behavioral dominance^29^. Thus, the large majority of individuals on queen removal nests exhibited perturbation of their caste-associated gene expression, even though only a few of these individuals exhibited responses to queen loss at the level of physiology or behavior.

Furthermore, we found no evidence that the degree of transcriptional perturbation declined over time following queen removal. SVM classification estimates of individuals from queen removal nests did not differ significantly between days three and twelve following queen removal (QR3 mean 0.331 ± 0.111; QR12 mean 0.345 ± 0.082; Wilcoxon *W* = 369, *p* = 0.55). The effects of queen removal on individuals’ gene expression profiles therefore appear to be both widespread and persistent, affecting all individuals in a nest and lasting beyond the point at which a new queen has already become phenotypically established.

Interestingly, the strong colony-wide perturbation following queen loss that this SVM approach identifies would have been entirely missed using a standard differential expression approach: a DESeq2 analysis with a 1.5-fold-change threshold identified just five genes as differentially expressed between control workers and individuals from manipulated nests, with no associated GO enrichment (Supplementary Data File 4). Even omitting the fold-change threshold, the number of genes identified as being differentially expressed between control and queen removal workers only rose to 291, a small fraction of the 2438 genes identified as differentially expressed between control queens and workers.

### Age and queenness explain variation in individual-level molecular responses to queen loss

Although SVM classification indicates that queen removal causes colony-wide perturbation to brain expression profiles, classifier estimates varied substantially between individuals following queen removal, spanning a much greater range of values (0.116–0.540) than those of queens (0.900–1.100) or workers (0.057–0.100) from queenright colonies (Fig. 2). To better understand this variation, we examined whether the classifier estimates for individuals from manipulated colonies were predicted by those individuals’ phenotypic traits—specifically ovarian development, behavioral dominance, and age^29^ (Fig. 1).

**Fig. 3:**
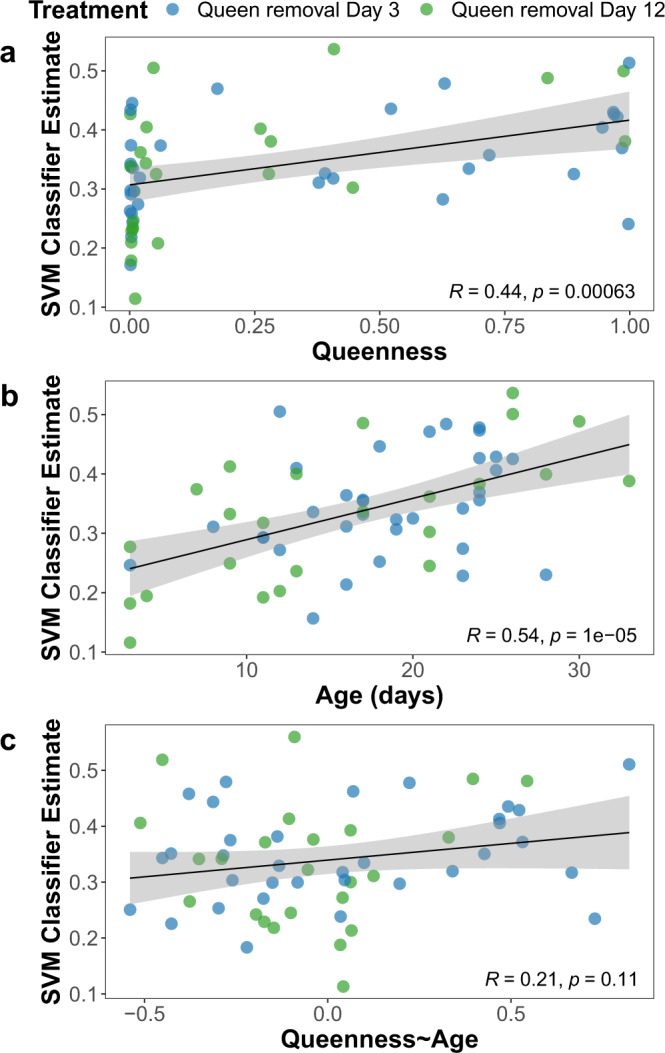
Correlation of SVM classifications with phenotypic traits among individuals from experimental colonies. Scatterplots of SVM classifier estimates for individuals from post-removal colonies (*n* = 58 biologically independent samples) plotted against **a** phenotypic queenness, **b** age, and **c** the residuals of queenness on age. *R* = Pearson correlation; *p* = uncorrected significance value of linear models fitting SVM classification against the specified variables. Regression line and 95% confidence interval for each model are shown in black and gray respectively.

Our phenotypic measure of caste identity (queenness) was a significant predictor of expression-based SVM classifier estimates when fitted using a linear model (slope±SE = 0.0414 ± 0.0114, *p* = 6.0 × 10^−4^; Fig. 3A). This relationship did not change when using queenness values recalculated using a phenotypic training set consisting of only the individuals for which gene expression data were generated (slope±SE = 0.0407 ± 0.0115, *p* = 8.3 × 10^−4^). The individual components of queenness, ovarian development and dominance were also significant or near-significant predictors of SVM classification individually (ovarian development: slope±SE = 0.0426 ± 0.0113, *p* = 4.0 × 10^−4^; dominance: slope±SE = 0.0231 ± 0.0122, *p* = 0.06). Notably, however, because phenotypic queenness was strongly correlated with age among post-removal individuals^29^ (cor = 0.4832, *p* = 8.0 × 10^−5^), age was an equally strong predictor of caste estimates when fitted in a separate linear model (slope±SE = 0.0516 ± 0.0106, *p* = 9.8 × 10^−6^; Fig. 3B). Thus, the significance of queenness as a predictor of caste estimates might have been an artifact of the fact that both are correlated with age. To test whether queenness had an effect over and above that accounted for by age, we calculated the residuals of phenotypic queenness on age and fitted the caste estimates against these. These residuals were not significantly predictive of SVM classification (slope±SE = 0.0202 ± 0.0123, *p* = 0.11; Fig. 3C), nor were the residuals of ovarian development alone on age (slope±SE = 0.0223 ± 0.0123, *p* = 0.07) or the residuals of dominance alone (slope±SE = 0.0114 ± 0.0126, *p* = 0.37). Age is thus the strongest determinant both of individuals’ caste phenotypes and of their caste-associated gene expression.

The control queens and workers that were used to train the SVM model differed not only in their caste, but also in their age—because foundress queens had overwintered, they were several months old at the time of sampling, whereas workers were weeks or days old. This fact, together with the observation that age predicts individuals’ SVM classifications, could suggest that our SVM model is in fact a classifier for age rather than caste. Yet, three lines of evidence speak against this possibility. First, control workers and queen removal individuals were of comparable age but their SVM classifications were clearly distinct (but should have been very similar if based purely on age). Second, age was not predictive of variation in the SVM classifications among control workers (slope±SE = 0.0054 ± 0.0033, *p* = 0.14). Third, the set of caste-informative genes identified by our SVM approach did not overlap significantly with a set of 625 age-biased genes identified using DESeq2 (Supplementary Data File 5; Jaccard index = 0.05; one-sided hypergeometric overlap *p* = 0.12). Taken together, these three results strongly suggest that the SVM model that we have produced classifies individuals by caste identity rather than by age.

In order to further discern the factors that shaped individuals’ gene expression profiles following queen removal, we performed weighted gene co-expression network analysis (WGCNA) using the full set of annotated genes. We generated 22 consensus modules across all samples, and then determined which traits significantly predicted the expression of a given module within each group (Supplementary Figs. S6–S8). Six modules exhibited a significant degree of overlap with the set of 1992 caste-predictive genes identified using SVM feature selection (Supplementary Fig. S9), including three modules whose expression was significantly correlated with either phenotypic traits or SVM classification in at least one treatment group.

Module 2 consists of 102 genes and appears to be associated with many caste-related traits in the queen removal condition, being negatively correlated with queenness, age, SVM classification and (less strongly) with ovarian development among queen removal individuals. We also found weak evidence that this module is negatively associated with age among workers from control nests. Unexpectedly, despite the seeming importance of Module 2 in predicting caste identity among workers, not a single GO term was enriched among the genes in the module at *p* < 0.01. A second module, Module 8, consists of 614 genes and has a strongly negative correlation with age among both control workers and queen removal individuals. This module is associated with 20 GO terms, including a number of terms associated with molecular binding, such as “protein binding”, “DNA binding”, and “RNA binding” (Supplementary Fig. S10).

Finally, Module 11 consists of 519 genes and is notable in that its expression in individuals from queen removal colonies is positively associated with SVM classifications but not with phenotypic correlates of caste—significantly, “chromatin remodeling” is one of the 22 GO terms with which this module is associated (Supplementary Fig. S11). Regulation of chromatin accessibility is one of several epigenetic processes that have been implicated in the control of caste expression in social insects^38–40^. The fact that Module 11 correlates with transcriptomic caste identity in post-removal nests might therefore suggest that disturbances to individuals’ gene expression patterns following queen removal reflect some form of epigenetic reprogramming^41^. Genes that were more strongly related to phenotypic queenness also exhibited higher module significance (connectivity) within Module 11 (cor = 0.6, *p* = 1.0 × 10^−78^), which was not the case for Module 2 (cor = 0.95, *p* = 0.34) or for Module 8 (cor = 0.077, *p* = 0.057). Taken together, these results strongly suggest a role for Module 11 in caste differentiation.

## Discussion

In this study, we have employed an SVM approach to interrogate the transcriptomic signatures of a complex plastic phenotype. Applying this approach to identify caste-specific gene expression profiles in *P. dominula* and to explore the relationship between transcriptomic and plastic phenotypic changes following a major social perturbation in paper wasp colonies, we identify a set of ~2000 genes that optimally capture gene expression differences between established queens and workers. Using a caste classifier based on these genes, we find that queen removal leads to a colony-wide shift in expression, where the expression profiles of all individuals move towards a state intermediate between those of established queens and workers. Individual variation around this intermediate state is related to age and phenotypic attributes, with older (and therefore more queen-like) individuals showing expression profiles that are closer to that expected for established queens. Our results show that molecular responses to queen removal in *P. dominula* consist of both a general colony-wide response independent of measured phenotypic change and a response that reflects the plastic phenotypic transition. Our findings also highlight the utility of SVMs, both to identify genes that reliably separate complex but well-defined phenotypes, and to identify transcriptomic shifts that occur when such phenotypes become plastic.

Our study contributes to the significant progress in our understanding of the relationship between molecular changes and changes in phenotypic expression that has been made in the past decade, facilitated by the increased availability of “omic” data and complex bioinformatic analyses. Recent studies have started to challenge the view that there is a direct correspondence between transcriptomic states and external phenotypes. Libbrecht et al.^15^, for example, show that gene expression responses associated with a reversible phenotypic change differ qualitatively based on the directionality of the change (from reproductive to non-reproductive or vice versa). Meanwhile, molecular manipulations have revealed a surprising degree of plasticity in canonically implastic traits such as mammalian sex^42^ or ant castes^13^. Our results appear to go further, showing a shift in caste-specific brain gene expression profiles among individuals whose phenotypic caste expression remains otherwise apparently unchanged. If accurate, this result shows that the expectation of a close match between expression profiles and phenotypes is excessively simplistic, or at least that detecting such a match requires detailed knowledge of relevant genes and/or exhaustive phenotypic characterization.

Although we failed to find a clear age-independent association between expression profiles and caste-related phenotypes, we cannot rule out the possibility that there are other, more subtle facets of caste identity in *P. dominula* that we failed to measure and that might explain variation in gene expression. For example, *Polistes* wasps are known to exhibit increased juvenile hormone titers following queen loss^20^, and foundress queens may possess more substantial lipid stores than early-season workers^43^. It is possible that changes in these traits or others would explain the large shifts in gene expression that we observed across all individuals following queen removal. We nonetheless consider it significant that the phenotypic changes associated with queen removal are not expressed at the level of ovarian development or dominance. These two traits are the ultimate determinants of caste identity: the fact that an individual has high JH levels does not matter to colony functioning if that same individual continues to occupy the social and reproductive role of a worker. Therefore, although we cannot unequivocally state that our results reflect a disconnect between phenotype and gene expression, it is certainly unexpected that changes in individuals’ caste-specific gene expression profiles would not be reflected at the level of caste expression that matters.

It is also possible that the relationship between caste phenotypes and expression profiles would be more obvious in tissues that were not assayed in our study (such as ovaries). We concentrated on the brain because we were able to assess individuals only over a relatively brief period following queen removal and this tissue is known to show high short-term expression plasticity^44,45^. Similarly, our choice to analyze transcriptomes of whole-brain tissue rather than singling out individual tissues within the brain was driven by both practical and scientific concerns: we aimed to acquire enough tissue per sample to avoid pooling, and also to remain agnostic regarding the specific regions of the brain that could be influential upon reproductive phenotypes. Despite the limitations of focusing on a single, heterogenous tissue type, our analyses revealed significant associations between measured expression patterns and ovarian development, indicating that brain gene expression at least partially reflects organismal physiology and the state of other tissue types.

A major advantage of this study is the use of individual-level gene expression data from a large number of subjects, including individuals reared in a shared social environment but exhibiting very different phenotypic responses to perturbation. By sequencing individuals rather than pools, we were able to match each gene expression profile to high-resolution phenotypic data that captures the scale of naturally occurring variation in features such as age, ovarian development, and dominance behavior. This resolution allows us to address questions that are otherwise inaccessible in gene expression analyses. For example, we have been able to show that caste identity, but not the residuals of caste identity on age, are significantly predictive of individuals’ change in transcriptomic caste identity following queen loss.

Our discovery of colony-wide responses to queen loss suggests that this social perturbation provokes a significant reaction even from individuals that have little hope of attaining the vacant reproductive role. This is an unexpected finding given that *P. dominula* is thought to express a “conventional” gerontocratic (age-based) mechanism of dominance and queen succession that mitigates the need for costly intragroup conflicts over the identity of the replacement queen^27,28,46^, which should greatly reduce the need for young, low-ranking workers to respond to queen loss^29^. The gene expression responses of lower-ranked workers to queen removal might plausibly represent a form of safeguard against queen loss: if queen loss sometimes occurs multiple times in quick succession or is frequently associated with a general reduction of the nest population (i.e., through predation), there might be kin-selected benefits of a colony-wide “de-differentiation” of individuals that facilitates a quicker succession process. This possibility is supported by the fact that one of the WGCNA modules that we identified was significantly enriched for the GO term “chromatin remodeling”. Regulation of chromatin accessibility, together with other epigenetic mechanisms, is a prime candidate mechanism for the regulation of caste identity in social insects^38–40^. The fact that this module was correlated with transcriptomic but not phenotypic caste identity among individuals from post-removal nests may therefore indicate that individuals respond to queen loss by priming their transcriptomes for further epigenetic changes, but that this priming only goes on to produce phenotypic effects if accompanied by a second trigger (such as the absence of an older sibling on the nest).

We identified 34 GO terms that were significantly enriched among the 1992 genes optimally separating queens and workers. Most of these terms were related to the regulation of gene expression or cell signaling, rather than to processes directly involved in reproduction or aggression. We also did not find a significant overlap between these 1992 genes and 1533 genes whose expression was correlated with vitellogenin in a previous study of caste in *P. dominula*^24^ (Jaccard index = 0.09; one-sided hypergeometric overlap *p* = 0.64), which reinforces the findings of our GO analysis because vitellogenin is thought to be a key regulator of aggression in *P. dominula*^26^. Although it might have been expected that GO terms associated with aggression or reproduction would be among the most highly enriched terms separating queens from workers in this study, this result does fit with previous studies of caste expression in *P. dominula*. For example, the most highly enriched GO terms differentiating queens and workers in Standage et al.^24^ were involved in biosynthetic processes, which the authors argue to be evidence in favor of the idea that highly conserved genes with basic biological functions may play a key role in the evolution of insect sociality (the “genetic toolkit” hypothesis^47^). Although our 1992 caste-biased genes do not significantly overlap with 295 genes that Standage et al identified as differentially expressed between queens and workers (Jaccard index = 0.02; one-sided hypergeometric overlap *p* = 0.72), the GO terms that we identified do correspond to highly conserved functions such as transcription and biosynthesis. Our results thus add to the increasing body of data supporting the genetic toolkit hypothesis.

Replacement queens in our colonies did not have access to males and therefore remained unmated even after queen succession. This may partially explain the fact that even individuals with fully developed ovaries and very high dominance ratings did not transition to a fully queen-like gene expression profile, as mating can induce significant gene expression changes in insects^48–50^. The lack of immediate mating opportunities for new queens in our experiment is not necessarily unrealistic, however: unmated hymenopteran females can lay unfertilized eggs, which develop as males. Moreover, in naturally occurring early *P. dominula* nests, replacement queens may be established a month or more before they are mated^19^, presumably owing to a scarcity of early males. The unmated replacement queens analyzed here are therefore representative of those that would be present on wild nests shortly after queen loss.

Applying a support vector classification approach to behavior-associated transcriptomic data, we identified a large group of genes as differing meaningfully between *Polistes* castes—over 10% of annotated genes, a similar number to that which we were able to identify using a standard differential expression program with no log fold-change threshold. Although the set of caste-biased genes identified by our SVM approach did not differ strongly in size from that generated using a standard approach, the use of SVMs nonetheless provides two clear advantages over standard analyses. First, SVM classification not only identified a set of genes that are predictive of caste identity, but the univariate classification that the model generated also allowed us to characterize the molecular caste identity of samples that did not fall squarely into the roles of control workers and queens. Standard differential expression analyses such as edgeR^51^, DESeq2^52^, or NOISeq^53^ assess differential expression at the level of individual genes. This focus makes these approaches well-suited to the identification and ranking of genes that distinguish pre-defined states, but of limited use when classifying intermediate or uncategorized samples, a purpose for which SVM analysis is ideally suited.

A second strength of the SVM classification approach is that it partially bypasses the requirement for judgements over which genes can be considered “biologically meaningful”. Differential expression studies almost always include a fold-change threshold in their analyses to ensure that they do not include genes with very small fold changes in expression that may be statistically but not biologically significant. Choice of fold-change threshold is largely arbitrary and can be hugely impactful upon the results achieved^54^. For example, in this study, we identified just 81 differentially expressed genes when using a 1.5-fold-change threshold, compared with 2438 genes when no threshold was applied. Both of these sets of genes exhibited reduced predictive ability compared with a model generated using SVM feature selection, suggesting that the standard differential expression approach either misses genes that are predictive of caste identity or includes genes that confound caste prediction (via overfitting). Indeed, the primary reason that the majority of standard differential expression analyses include a fold-change threshold is a concern that failure to do so will result in the identification of genes that are statistically differentially expressed but are biologically uninformative^55^. Our results reinforce this notion. By contrast, the decision of whether a given gene was included in our SVM model was based on a criterion that directly reflects biological relevance: did expression measures for that gene provide additional information about an individual’s likely caste identity, and hence improve the predictive ability of our model?

Here we have undertaken a detailed analysis of the relationship between gene expression and socially mediated phenotypic plasticity, revealing broad-scale changes in caste-associated gene expression profiles following a major social disruption. Our findings demonstrate the value of SVMs both to generate sets of genes that meaningfully predict differences between well-defined phenotypes and to identify the transcriptomic changes that accompany subsequent plasticity in such phenotypes. We reveal a hitherto unrecognized capacity for large-scale disruption to caste-biased gene expression profiles even in the absence of apparent changes in caste phenotype, a disconnect that undermines simplistic models of the relationship between transcriptome and phenotype. Future studies should continue to marry detailed phenotypic and gene expression data in order to assess the prevalence and provenance of such discontinuities.

## Methods

### Sample collection

*Polistes dominula* colonies (*n* = 72) were collected from rural areas near Florence, Italy. Nests were transferred to the laboratory prior to the eclosion of workers and subordinate foundresses were removed. After at least 4 workers had eclosed, nests were filmed for 3 days and then assigned to either control or queen removal treatments. Following treatment (sham or queen removal) nests were filmed for a further three or twelve days and then all individuals were dissected and measured using ImageJ^56^ to generate ovarian development indices^57^. Footage of nests was annotated using BORIS behavioral annotation software^58^ and then used to generate dominance indices in the form of Elo ratings^59,60^. Ovarian and Elo rating data were fed into a Bayesian logistic model to produce a caste classifier, which we then used to produce a holistic measure of caste identity (“queenness”) for individuals from queen removal nests. A queenness estimate of 1 indicates that an individual is identical to a control queen in terms of ovarian development and behavioral dominance, whereas a value of 0 indicates complete identity to a control worker by the same measures. Using these methods, ovarian, dominance, and queenness data were generated for 220 workers from control nests, 330 individuals from queen removal nests and 54 queens. Complete details of all the above methods can be found in Taylor et al.^29^.

From among these individuals we randomly selected 29 queens and 20 control workers for sequencing. We additionally selected 65 individuals from queen removal nests for sequencing, using stratified random sampling to cover a full range of values of ovarian development, Elo rating, and queenness (Supplementary Data File 6). A number of these samples failed at the point of transcriptome sequencing (next section), leaving us with final sample sizes of 12 control workers, 58 queen removal individuals, and 26 queens.

### Gene expression quantification

Brain tissue was extracted from the heads of individual samples and RNA was extracted using the RNeasy Mini Kit (Qiagen) according to manufacturer’s instructions. Library preparation was performed by Novogene Co. followed by sequencing on an Illumina HiSeq 2000 platform with 150-base pair paired-end reads. Reads were filtered with SortMeRNA^61^ using default options to remove ribosomal sequences. Trimmomatic^62^ was then used to perform quality trimming. First, we trimmed adapter sequences and leading and trailing bases with low phred scores (<3). We then used the MAXINFO option with target length 36 and strictness 0.7 to trim low-quality sequences from the remaining reads. Reads were next mapped to 11,313 transcripts from the *P. dominula* genome annotation 1.0^24^ using STAR^63^ with default options. All 101 samples produced >85% uniquely mapped reads. Reads were assembled into transcripts using StringTie2^64^ before being passed on as raw counts to downstream analyses. Between each step, the quality of reads for each sample was checked using FastQC^65^. Prior to downstream analysis, transcripts were filtered to remove any gene which did not have >20 counts across all samples in at least one of the experimental groups (queens, control workers, and day 3 and day 12 post-manipulation workers). Following this filtering, 10734/11,313 (94.9%) of transcripts remained.

### SVM classification

Support vector classification was performed in R^66^ using the e1071 package^67^ using the gene expression profiles for all available queens (coded with a value of 1; *n* = 26) and control workers (coded with a value of 0; *n* = 12). Classifiers were assessed via their threefold cross-validation error rates; classifiers with lower classification error were considered to be superior. SVM classification operates by taking multidimensional data (e.g., the expression levels of many different genes) pertaining to two or more classes (e.g., queens and workers) and identifying a kernel function that will transform the data such that the classes can be linearly separated. The choice of kernel function is optimized by performing a “grid search” in which each combination of parameters within the kernel function is tested across a range of values in order to identify the set of parameters that best allow the data to be linearly separated between classes. Initially, we tested classifiers using a variety of kernel functions—radial, linear, sigmoid, and polynomial—combined with grid searches across a wide range of parameters for each kernel (kernel parameters vary depending on the form of the kernel, but always include a cost parameter *C*, which determines how strongly misclassifications are penalized). The kernel function that produced the lowest error rate was the radial function, which gives the distance between two samples *x* and *y* as:

1 $${\mathrm{exp}}\left( { - \gamma \left| {x - y} \right|^2} \right)$$

A radial kernel with *γ* = 10^−6^ and cost parameter *C* = 2^5^ was found to produce the lowest error rate of all combinations of parameters, so all subsequent classifiers were fit using this kernel and a more focused grid search in a parameter space of 2^4^ < *C* < 2^6^ and 10^−5^ < *γ* < 10^−7^ in order to minimize the processing power necessary to perform feature selection. For feature selection, we took the classifier fitted with all genes, and iteratively performed the following process: (1) the threefold cross-validation error of the model was calculated twenty times using randomly assigned bins, and the mean of the resulting errors was recorded as the true validation error of the classifier; (2) the feature weights of all genes were calculated by taking the matrix product of that classifier’s coefficients with its support vectors; (3) the gene with the smallest absolute weight in the model was dropped; (4) a new classifier was calculated using the remaining set of genes. This process was repeated until just 100 genes remained, and the optimal support vector classifier was then taken as that for which the cross-validation error reached its minimum.

### Differential expression analysis

Differential expression analyses were performed in R using the DESeq2 package^52^. DESeq2 was run on all groups and contrasts were then calculated for each pair of groups. Unless otherwise stated, differential expression was calculated relative to a baseline fold change of 1.5, i.e., *p* values refer to the probability that absolute change between two groups was >50%. Genes were considered differentially expressed between conditions if *p* < 0.05 after false discovery rate correction according to the Benjamini–Hochberg procedure.

### Gene co-expression network analysis

WGCNA was performed in R using the WGCNA package^68^. As WGCNA is particularly sensitive to genes with low expression, data were first subjected to a second round of filtering in which genes that had <10 reads in >90% of samples were removed, as recommended by the package authors. This second round of filtering removed an additional 1631 genes, leaving a total of 9103 genes. Counts were then subjected to a variance-stabilizing transformation prior to further analysis, Consensus gene modules across all samples were then constructed using a soft-threshold power of 9. Initially, 26 gene modules were identified. Modules whose eigengene correlation was >0.75 were subsequently merged, after which 22 consensus modules remained. Finally, the Pearson correlation of each module with each phenotypic trait within each group (queens, control workers, and individuals from queen removal nests) was calculated and subjected to Benjamini–Hochberg FDR correction. Network summary measures and gene dendrograms for WGCNA are provided in Supplementary Figs. S12–13.

### GO enrichment analysis

In order to perform GO enrichment analysis, we first used OrthoFinder^69^ to identify orthologues for each *P. dominula* gene in *Drosophila melanogaster*, a model species for which GO annotations are much more complete. GO annotations for each *D. melanogaster* gene were acquired from BioMart^70^ and each *P. dominula* gene was then assigned GO terms permissively, i.e., a given *P. dominula* gene was assigned a GO term if that term appeared as an annotation to any of its orthologues. 6659/10734 (62.0%) of genes possessed at least one orthologue in *D. melanogaster*. GO enrichment analysis was then performed in R via the topGO package^71^ using topGO’s weight01 algorithm and Fisher’s exact test to identify GO terms that were significantly overrepresented (*p* < 0.01) in a focal set of genes against a background consisting of all genes that appeared in the relevant analysis.

### Reporting summary

Further information on research design is available in the Nature Research Reporting Summary linked to this article.

## Supplementary information

Supplementary Information

Peer Review File

Reporting Summary

Description of Additional Supplementary Files

Supplementary Data 1

Supplementary Data 2

Supplementary Data 3

Supplementary Data 4

Supplementary Data 5

Supplementary Data 6

## Data Availability

Sequencing data associated with this paper have been deposited in the NCBI Gene Expression Omnibus with accession number GSE153532. Source data are provided with this paper.

## Source data

Source Data

## Code availability

Custom R code used to perform support vector classification and feature selection are provided in a GitHub repository [10.5281/zenodo.4390131], with instructions for use.
