# Supplementary material for: The molecular basis of socially mediated phenotypic plasticity in a eusocial paper wasp
Source: Nat Commun. 2021 Feb 3;12:775. doi: 10.1038/s41467-021-21095-6 (PMC7859208; doi:10.1038/s41467-021-21095-6)

Supplementary materials to Taylor et al (2021) *The molecular basis of socially-mediated phenotypic plasticity in a eusocial paper wasp*

Figure S1. Root mean squared three-fold cross-validation error of SVMs trained using an iteratively decreasing number of genes. In each iteration, the lowest-weight gene of the previous model was removed and a new model was trained with the remaining genes. For each model, three-fold cross validation error was taken as the mean of 20 calculations using randomly-selected validation bins. The error shown in figure is a moving average with a window size of 50. Red dashed line shows the minimum validation error achieved.

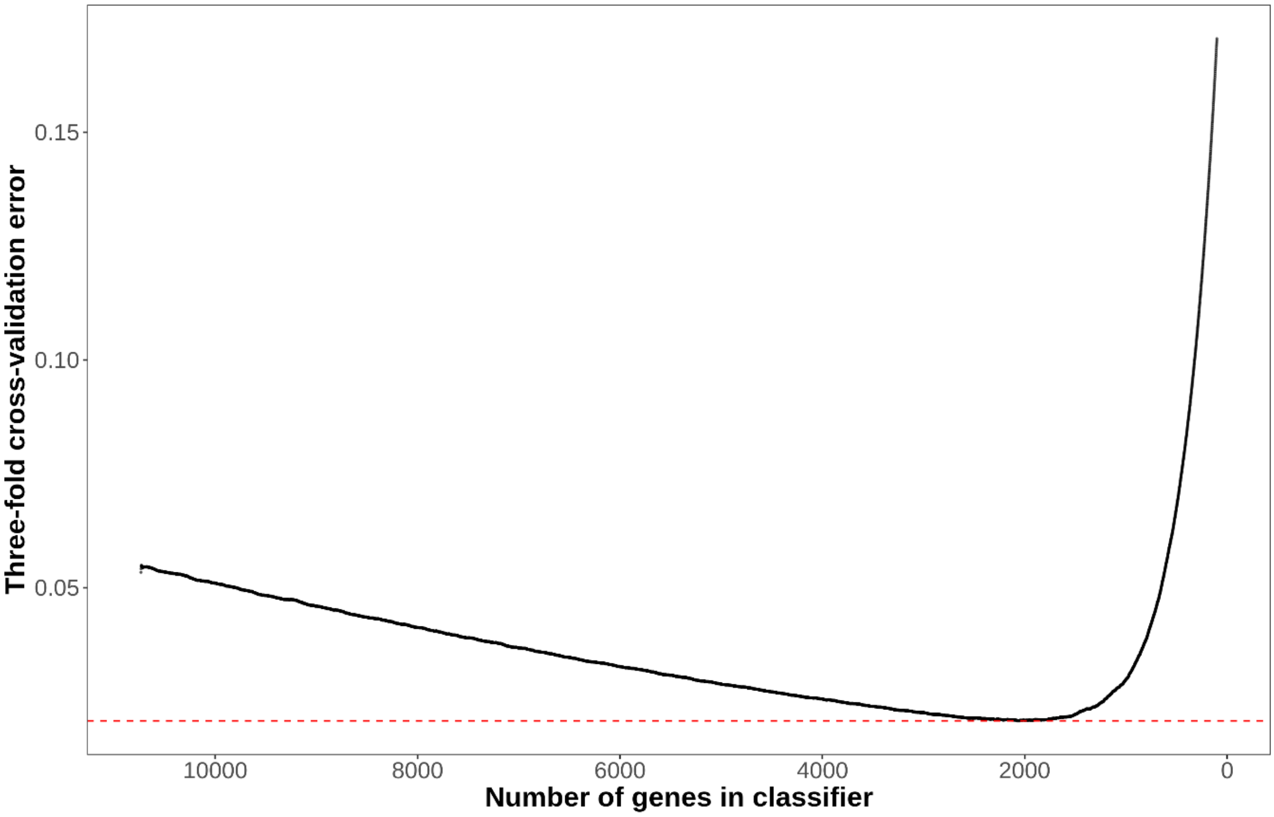

Fig S2. Significantly enriched gene ontology terms at p<0.01 among 1992 caste-informative genes identified by SVM classification.

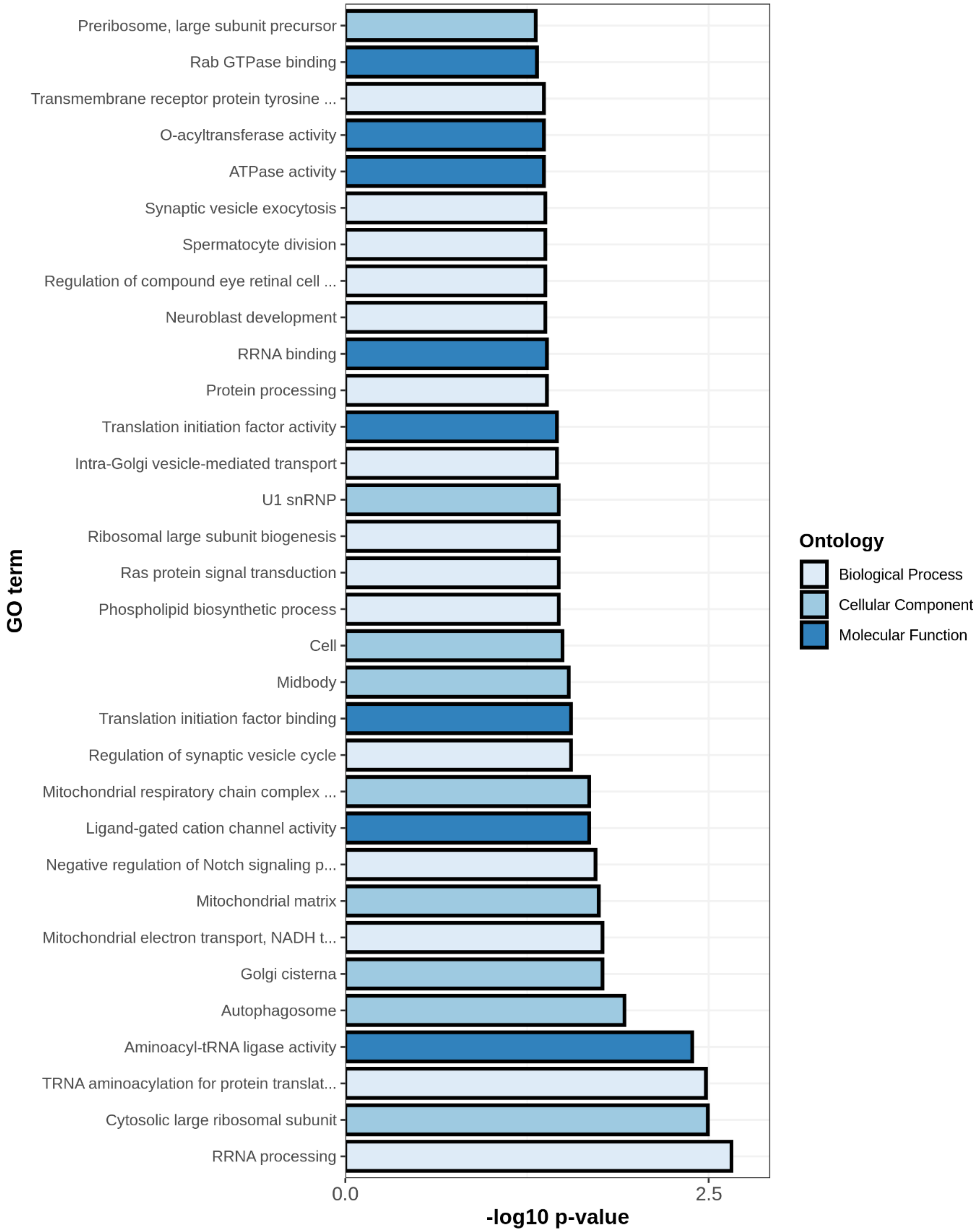

Fig S3. Absolute feature weights of the 1992 genes in the optimised SVM. 81 genes identified by DESeq2 as being differentially expressed between queens and control workers with a baseline log2 fold-change of 1.5 are marked in blue.

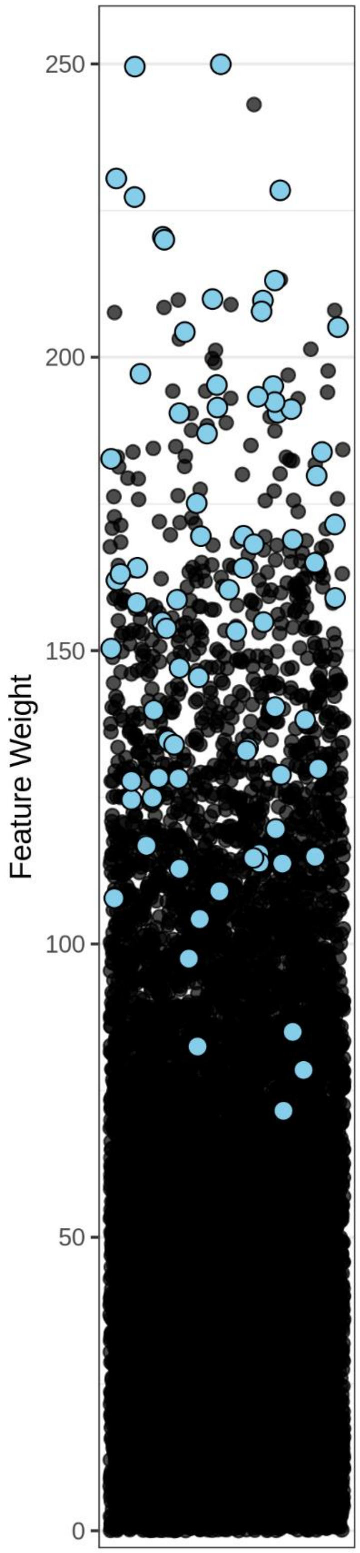

Fig S4. Distribution of phenotypic traits of sequenced individuals from queen removal colonies. Where possible, individuals were selected to represent as wide as possible a range of values for (A) Ovarian development; (B) Dominance; and (C) Phenotypic caste identity ('queenness'). Full methodology for the calculation of phenotypic indices is given in Taylor et al (2020).

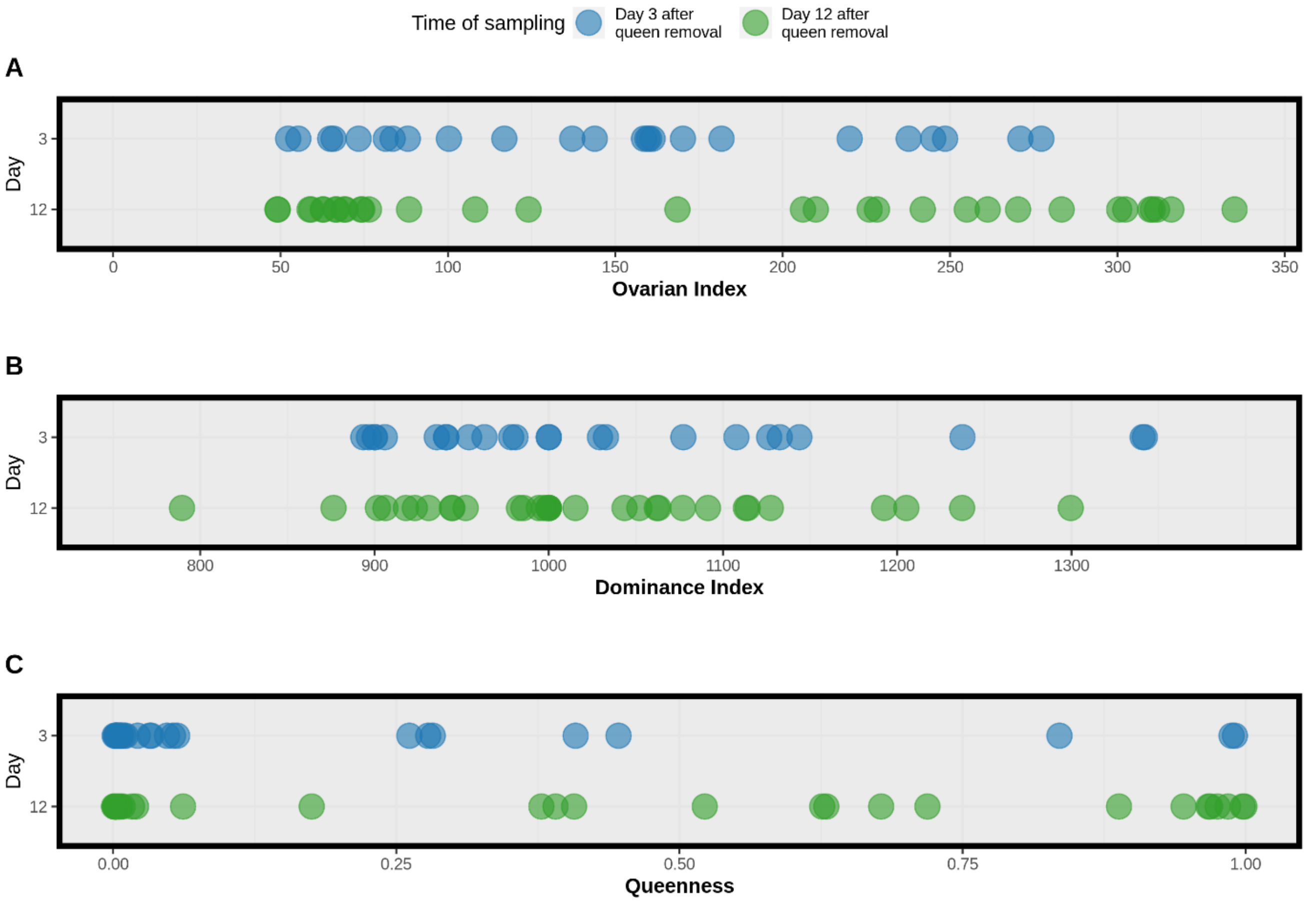

Fig S5. First two principal components generated by PC analysis of 1992 caste-associated genes identified by SVM classification.

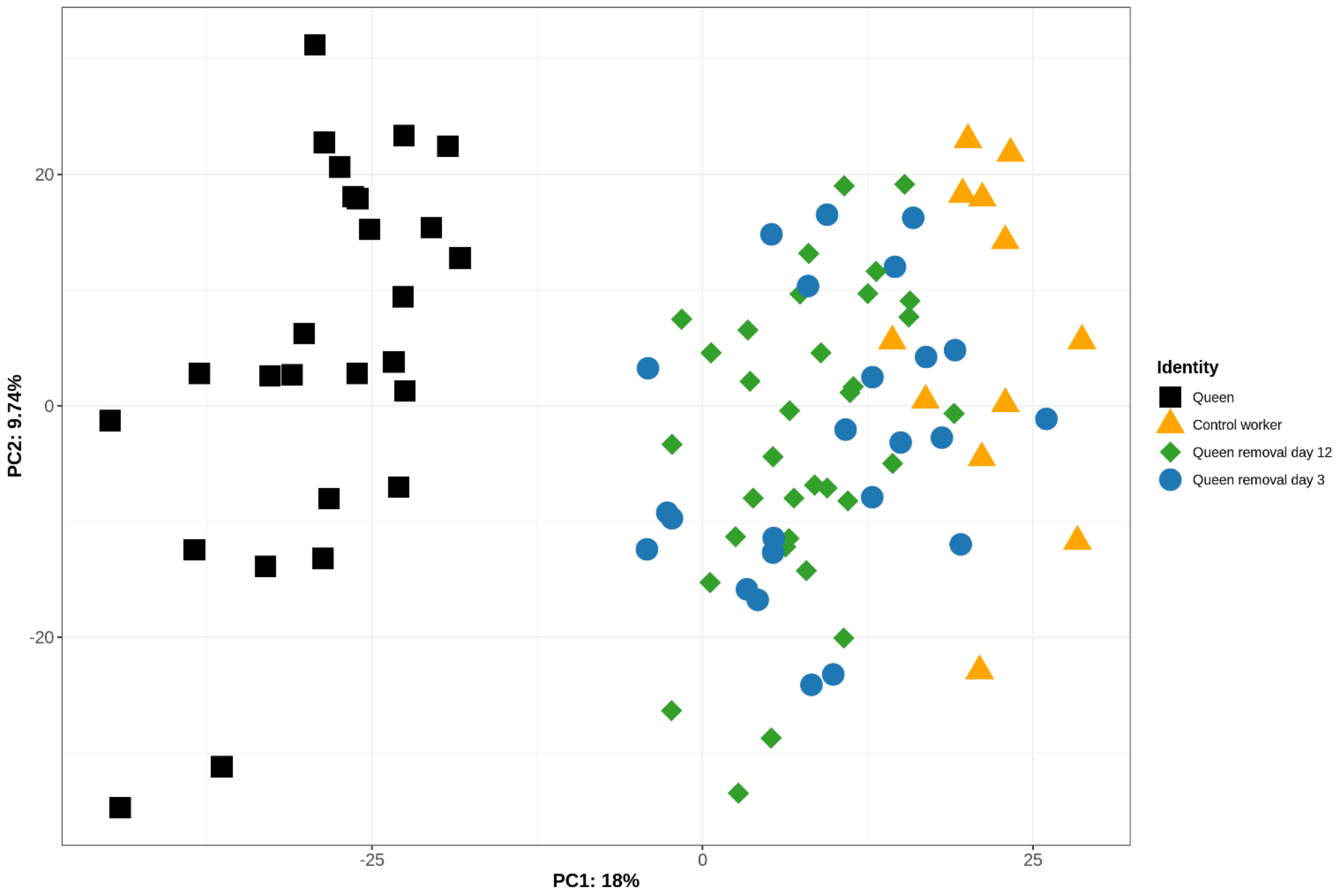

Fig S6. Module-trait correlations for consensus modules identified by WGCNA in individuals from queen removal nests (n = 62). Within each cell, the Pearson correlation coefficient is given above and the FDR-adjusted p-value is given below in parentheses.

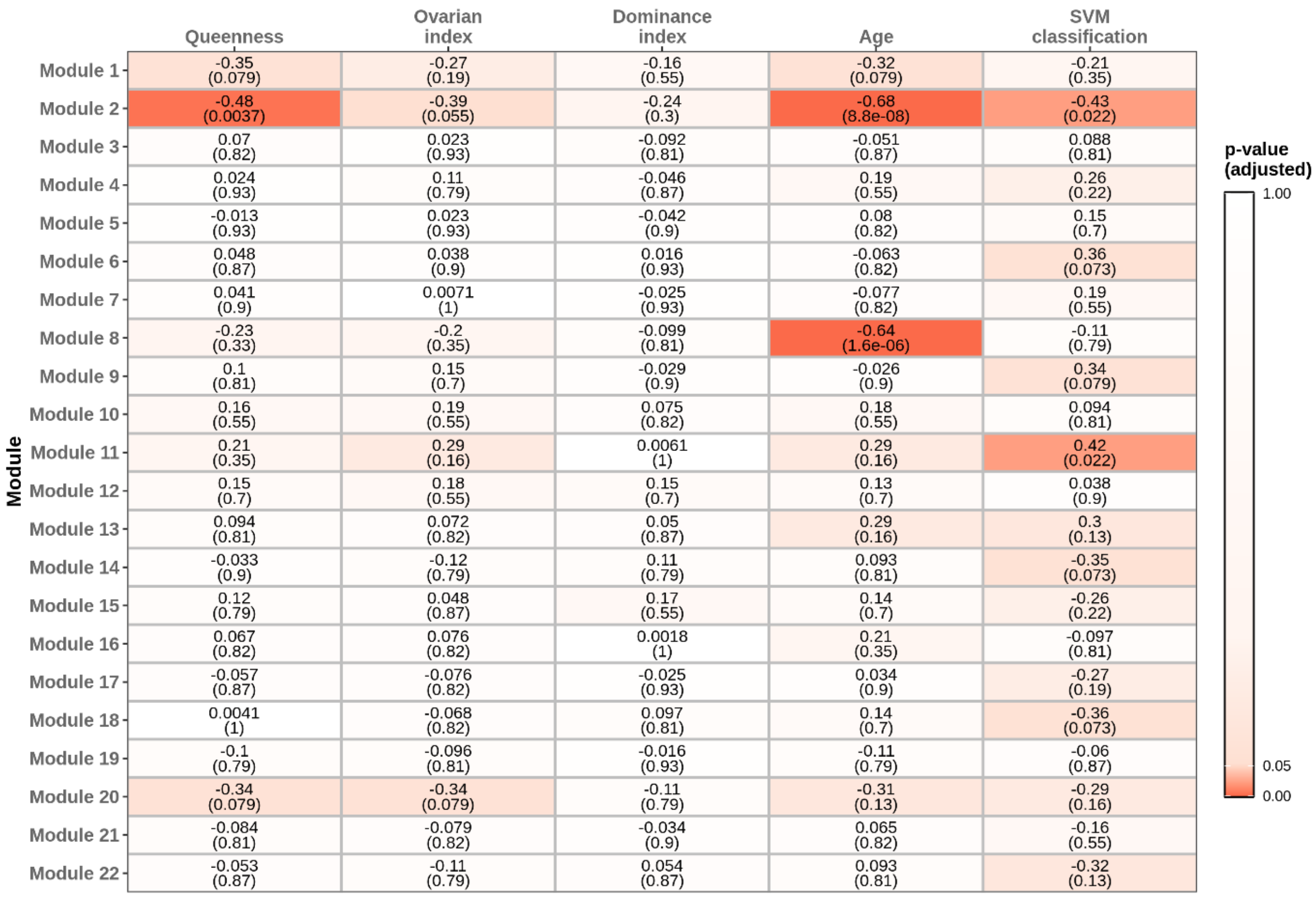

Module	Queenness	Ovarian index	Dominance index	Age	SVM classification
Module 1	-0.35 (0.079)	-0.27 (0.19)	-0.16 (0.55)	-0.32 (0.079)	-0.21 (0.35)
Module 2	-0.48 (0.0037)	-0.39 (0.055)	-0.24 (0.3)	-0.68 (8.8e-08)	-0.43 (0.022)
Module 3	0.07 (0.82)	0.023 (0.93)	-0.092 (0.81)	-0.051 (0.87)	0.088 (0.81)
Module 4	0.024 (0.93)	0.11 (0.79)	-0.046 (0.87)	0.19 (0.55)	0.26 (0.22)
Module 5	-0.013 (0.93)	0.023 (0.93)	-0.042 (0.9)	0.08 (0.82)	0.15 (0.7)
Module 6	0.048 (0.87)	0.038 (0.9)	0.016 (0.93)	-0.063 (0.82)	0.36 (0.073)
Module 7	0.041 (0.9)	0.0071 (1)	-0.025 (0.93)	-0.077 (0.82)	0.19 (0.55)
Module 8	-0.23 (0.33)	-0.2 (0.35)	-0.099 (0.81)	-0.64 (1.6e-06)	-0.11 (0.79)
Module 9	0.1 (0.81)	0.15 (0.7)	-0.029 (0.9)	-0.026 (0.9)	0.34 (0.079)
Module 10	0.16 (0.55)	0.19 (0.55)	0.075 (0.82)	0.18 (0.55)	0.094 (0.81)
Module 11	0.21 (0.35)	0.29 (0.16)	0.0061 (1)	0.29 (0.16)	0.42 (0.022)
Module 12	0.15 (0.7)	0.18 (0.55)	0.15 (0.7)	0.13 (0.7)	0.038 (0.9)
Module 13	0.094 (0.81)	0.072 (0.82)	0.05 (0.87)	0.29 (0.16)	0.3 (0.13)
Module 14	-0.033 (0.9)	-0.12 (0.79)	0.11 (0.79)	0.093 (0.81)	-0.35 (0.073)
Module 15	0.12 (0.79)	0.048 (0.87)	0.17 (0.55)	0.14 (0.7)	-0.26 (0.22)
Module 16	0.067 (0.82)	0.076 (0.82)	0.0018 (1)	0.21 (0.35)	-0.097 (0.81)
Module 17	-0.057 (0.87)	-0.076 (0.82)	-0.025 (0.93)	0.034 (0.9)	-0.27 (0.19)
Module 18	0.0041 (1)	-0.068 (0.82)	0.097 (0.81)	0.14 (0.7)	-0.36 (0.073)
Module 19	-0.1 (0.79)	-0.096 (0.81)	-0.016 (0.93)	-0.11 (0.79)	-0.06 (0.87)
Module 20	-0.34 (0.079)	-0.34 (0.079)	-0.11 (0.79)	-0.31 (0.13)	-0.29 (0.16)
Module 21	-0.084 (0.81)	-0.079 (0.82)	-0.034 (0.9)	0.065 (0.82)	-0.16 (0.55)
Module 22	-0.053 (0.87)	-0.11 (0.79)	0.054 (0.87)	0.093 (0.81)	-0.32 (0.13)

Fig S7. Module-trait correlations for consensus modules identified by WGCNA in workers from control nests (n = 12). Within each cell, the Pearson correlation coefficient is given above and the FDR-adjusted p-value is given below in parentheses.

Module	Ovarian index	Dominance index	Age
Module 1	-0.62 (0.2)	-0.42 (0.37)	-0.51 (0.25)
Module 2	-0.72 (0.11)	-0.58 (0.22)	-0.76 (0.082)
Module 3	-0.64 (0.18)	-0.39 (0.37)	-0.45 (0.37)
Module 4	-0.58 (0.22)	-0.1 (0.85)	-0.53 (0.25)
Module 5	-0.6 (0.22)	-0.14 (0.77)	-0.54 (0.25)
Module 6	0.37 (0.45)	0.28 (0.55)	-0.19 (0.69)
Module 7	-0.3 (0.55)	0.059 (0.91)	-0.18 (0.69)
Module 8	-0.48 (0.25)	-0.35 (0.45)	-0.86 (0.02)
Module 9	-0.66 (0.13)	-0.39 (0.37)	-0.71 (0.11)
Module 10	0.46 (0.37)	0.17 (0.69)	0.61 (0.22)
Module 11	0.33 (0.45)	0.23 (0.67)	0.79 (0.066)
Module 12	0.47 (0.25)	0.35 (0.45)	0.67 (0.13)
Module 13	0.43 (0.37)	0.061 (0.91)	0.75 (0.082)
Module 14	0.66 (0.13)	0.38 (0.45)	0.62 (0.2)
Module 15	0.53 (0.25)	0.2 (0.69)	0.67 (0.13)
Module 16	0.58 (0.22)	0.34 (0.45)	0.5 (0.25)
Module 17	0.44 (0.37)	0.16 (0.69)	0.17 (0.69)
Module 18	0.53 (0.25)	0.26 (0.55)	0.39 (0.37)
Module 19	0.19 (0.69)	0.0037 (1)	-0.092 (0.85)
Module 20	-0.4 (0.37)	-0.3 (0.55)	-0.45 (0.37)
Module 21	-0.15 (0.77)	-0.044 (0.91)	-0.37 (0.45)
Module 22	0.13 (0.77)	0.35 (0.45)	-0.19 (0.69)

p-value (adjusted): 1.00, 0.05, 0.00

Fig S8. Module-trait correlations for consensus modules identified by WGCNA in queens from control nests (n = 27). Within each cell, the Pearson correlation coefficient is given above and the FDR-adjusted p-value is given below in parentheses.

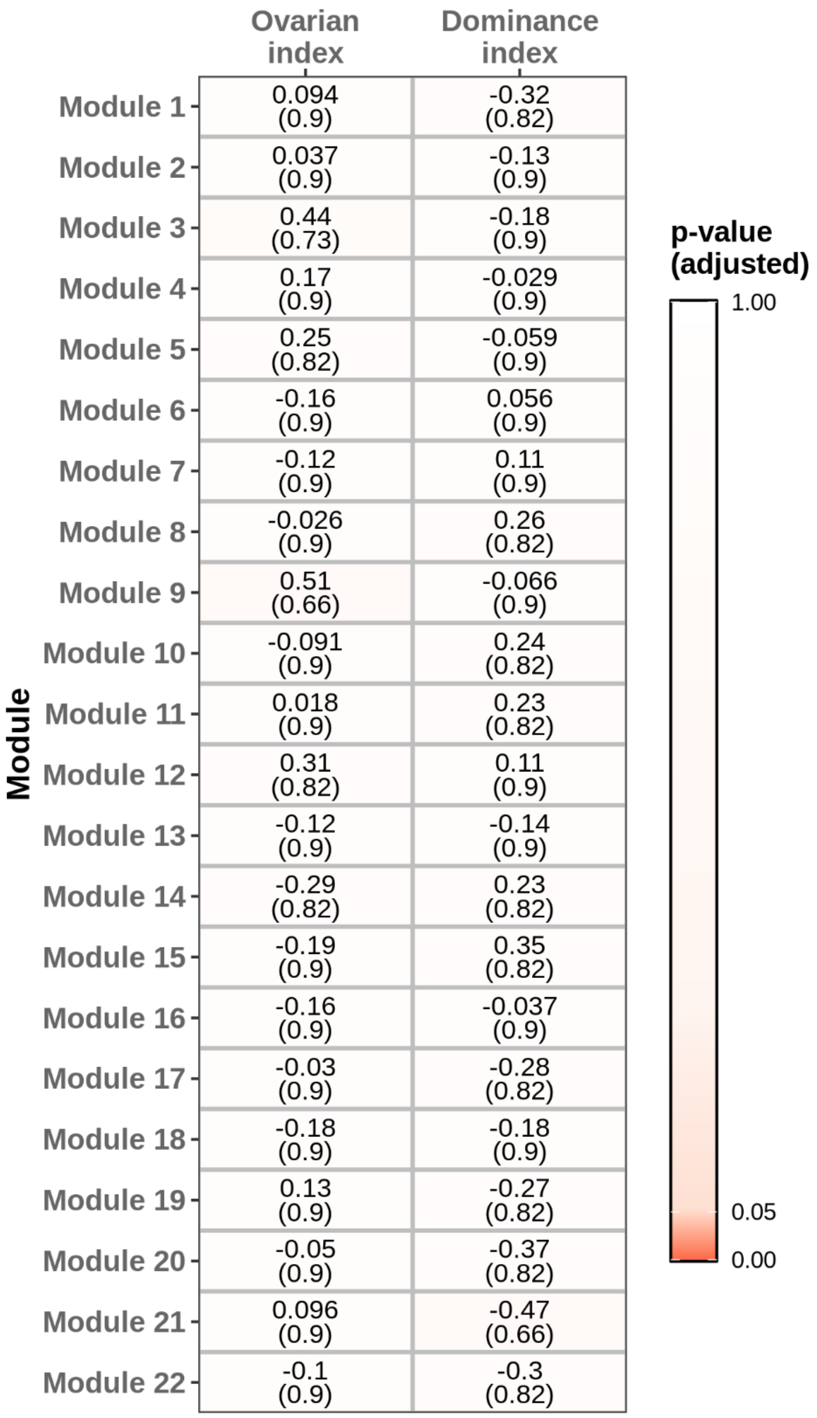

Figure S9. Overlap of gene modules identified by WGCNA and the set of caste-predictive genes identified by SVM feature selection. **Hypergeometric p**: p-value of a hypergeometric test assessing the overlap of the set of genes in the given module with the set of SVM genes. Where $p<0.05$, cells are shaded red. **Jaccard index**: The Jaccard similarity coefficient for the genes in the module with the genes in the SVM set. 1 = perfect overlap; 0 = no overlap.

Module	Hypergeometric *p*	Jaccard index
01	0.99626	0.032
02	0.00099	0.016
03	0.97079	0.020
04	0.49369	0.046
05	0.99844	0.024
06	0.10656	0.038
07	0.45331	0.024
08	0.04759	0.053
09	0.80162	0.008
10	0.00480	0.095
11	0.04126	0.064
12	0.07294	0.012
13	0.99459	0.001
14	0.99189	0.059
15	0.81359	0.044
16	0.04809	0.079
17	0.97796	0.005
18	0.72614	0.007
19	0.00042	0.023
20	0.95695	0.003
21	0.25200	0.017
22	0.73293	0.007

Fig S10. Significantly enriched gene ontology terms at p<0.01 for gene module 8.

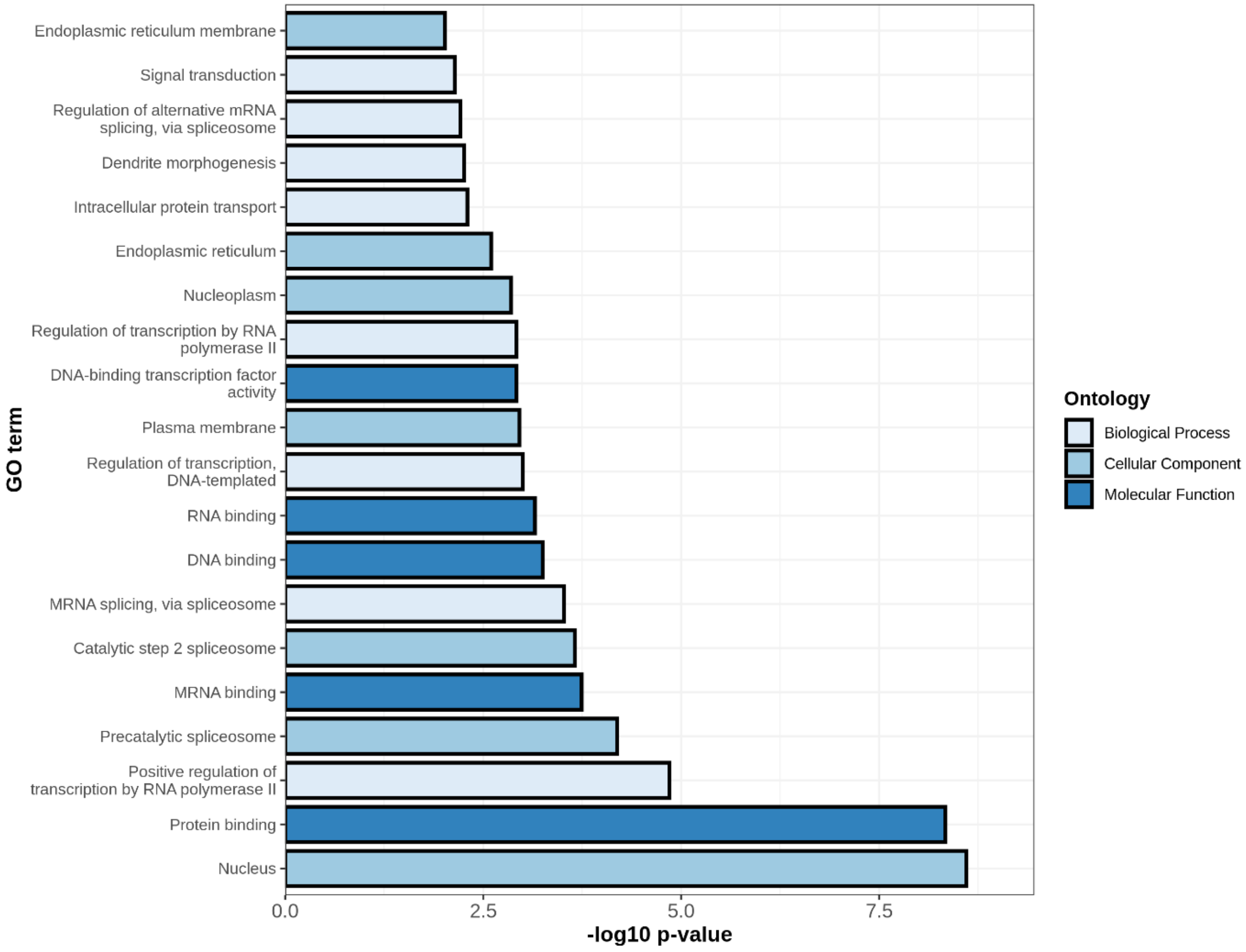

Fig S11. Significantly enriched gene ontology terms at p<0.01 for gene module 11.

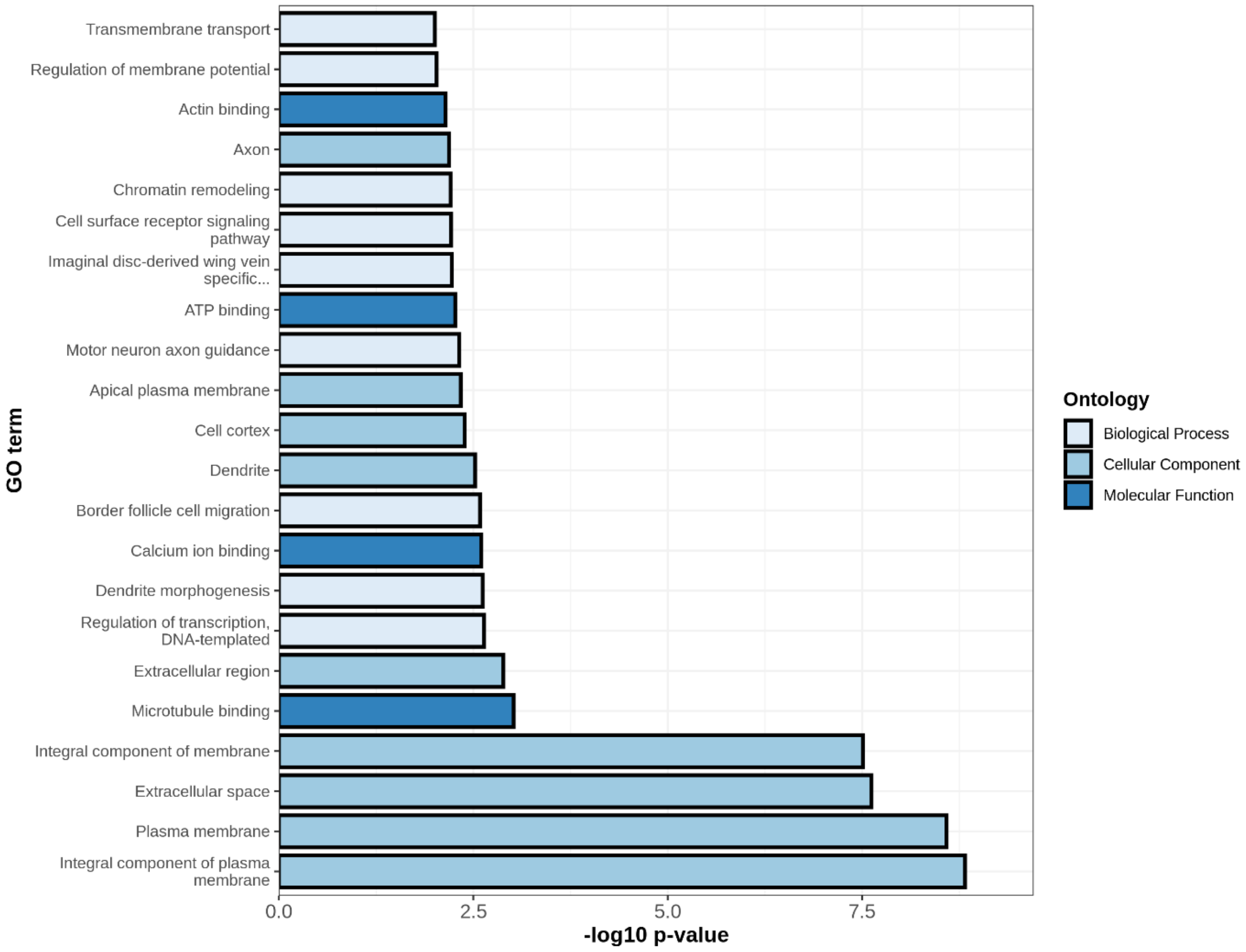

Fig S12. WGCNA summary network measures for each treatment group against soft thresholding power. Numbers in the plots indicate the corresponding soft thresholding powers. A soft thresholding power of 9 was chosen to balance model fit against connectivity.

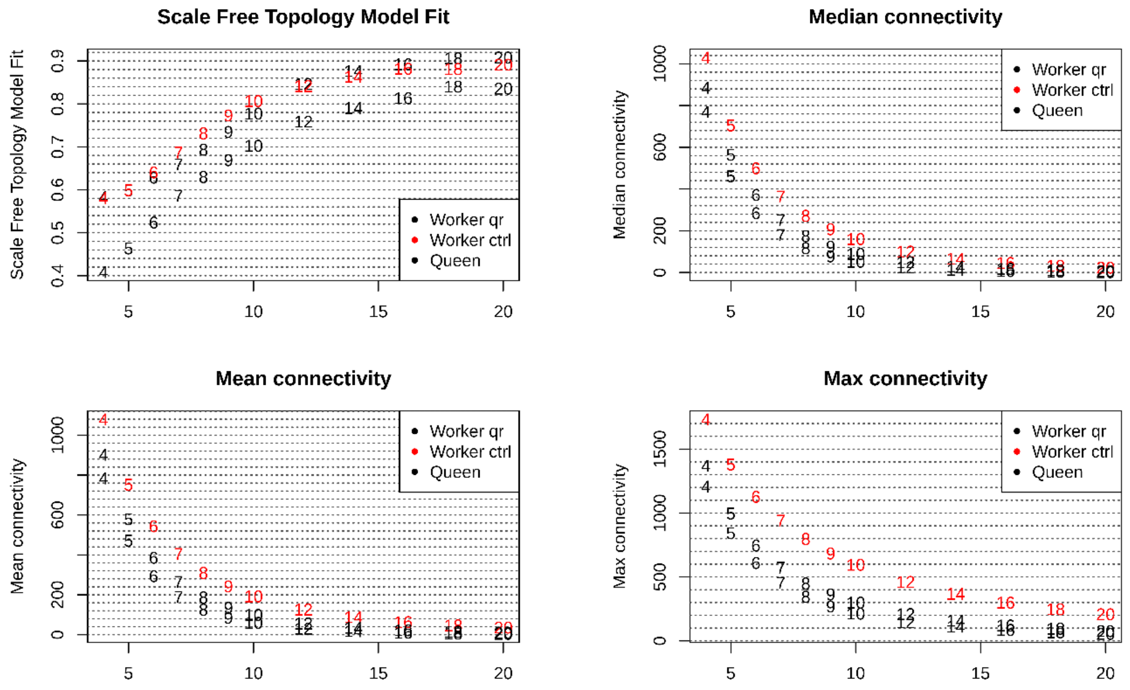

Fig S13. Gene dendrogram with clustering based on consensus topological overlap. Upper colour row: consensus module assignments prior to merging of modules with similar expression profiles. Lower colour row: consensus modules following merging of similar modules.

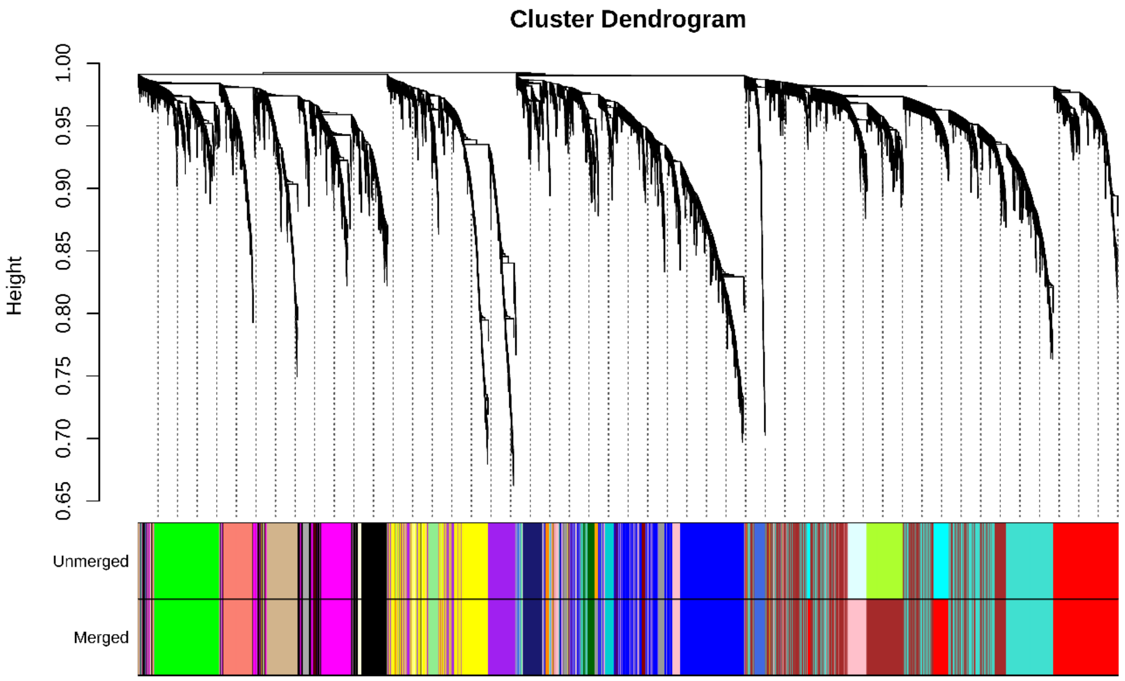

Supplement: Supplementary file 1 — Supplementary Information [file 41467_2021_21095_MOESM1_ESM.pdf]
