# Supplementary material for: The molecular basis of socially mediated phenotypic plasticity in a eusocial paper wasp
Source: Nat Commun. 2021 Feb 3;12:775. doi: 10.1038/s41467-021-21095-6 (PMC7859208; doi:10.1038/s41467-021-21095-6)
Supplement: Supplementary file 3 — Reporting Summary [file 41467_2021_21095_MOESM3_ESM.pdf]

nature research

Corresponding author(s): Benjamin Aaron Taylor

Last updated by author(s): 2020/12/24

Reporting Summary

Nature Research wishes to improve the reproducibility of the work that we publish. This form provides structure for consistency and transparency in reporting. For further information on Nature Research policies, see our Editorial Policies and the Editorial Policy Checklist.

Statistics

For all statistical analyses, confirm that the following items are present in the figure legend, table legend, main text, or Methods section.

n/a	Confirmed	
☐	☒	The exact sample size (n) for each experimental group/condition, given as a discrete number and unit of measurement
☐	☒	A statement on whether measurements were taken from distinct samples or whether the same sample was measured repeatedly
☐	☒	The statistical test(s) used AND whether they are one- or two-sided<br>*Only common tests should be described solely by name; describe more complex techniques in the Methods section.*
☐	☒	A description of all covariates tested
☐	☒	A description of any assumptions or corrections, such as tests of normality and adjustment for multiple comparisons
☐	☒	A full description of the statistical parameters including central tendency (e.g. means) or other basic estimates (e.g. regression coefficient) AND variation (e.g. standard deviation) or associated estimates of uncertainty (e.g. confidence intervals)
☐	☒	For null hypothesis testing, the test statistic (e.g. F, t, r) with confidence intervals, effect sizes, degrees of freedom and P value noted<br>*Give P values as exact values whenever suitable.*
☒	☐	For Bayesian analysis, information on the choice of priors and Markov chain Monte Carlo settings
☒	☐	For hierarchical and complex designs, identification of the appropriate level for tests and full reporting of outcomes
☐	☒	Estimates of effect sizes (e.g. Cohen's d, Pearson's r), indicating how they were calculated

Our web collection on statistics for biologists contains articles on many of the points above.

Software and code

Policy information about availability of computer code

Data collection	Boris v7.0.12<br>ImageJ v1.52
Data analysis	R v4.0.2<br>SortMeRNA v2.1<br>Trimmomatic v0.39<br>STAR v2.7<br>StringTie2 v1.3.6<br>OrthoFinder v2.3.7<br>FastQC v0.11.8<br>BioMart v2.46.0<br><br>GitHub with custom R code: https://github.com/BenjaminATaylor/Taylor-et-al-2020-demo

For manuscripts utilizing custom algorithms or software that are central to the research but not yet described in published literature, software must be made available to editors and reviewers. We strongly encourage code deposition in a community repository (e.g. GitHub). See the Nature Research guidelines for submitting code & software for further information.

Data

Policy information about availability of data

All manuscripts must include a data availability statement. This statement should provide the following information, where applicable:

- Accession codes, unique identifiers, or web links for publicly available datasets
- A list of figures that have associated raw data
- A description of any restrictions on data availability

Sequencing data associated with this paper have been deposited in the NCBI Gene Expression Omnibus (www.ncbi.nlm.nih.gov/geo) under accession number GSE153532 and are now publicly available.

Field-specific reporting

Please select the one below that is the best fit for your research. If you are not sure, read the appropriate sections before making your selection.

[x] Life sciences   [ ] Behavioural & social sciences   [ ] Ecological, evolutionary & environmental sciences

For a reference copy of the document with all sections, see nature.com/documents/nr-reporting-summary-flat.pdf

Life sciences study design

All studies must disclose on these points even when the disclosure is negative.

Sample size	Target sample sizes for each control groups were set at ~20 for queens and control workers and ~25-35 for the two groups of queen removal individuals. Sample sizes were selected to be well above the standard for our field (in which gene expression data rarely exceed 10 replicates/group, e.g. Warner et al Nature 2019 use three biological replicates/group; Weiner et al Genome 2017 use 5-7 biological replicates/group), to account for the additional noise generated by sequencing individuals rather than pools. Sample sizes for queen removal individuals were much larger than those for queens and control workers, because queen removal individuals exhibit much greater phenotypic heterogeneity. The sample sizes were chosen to allow us to identify expression correlates of this heterogeneity within cost constraints.
Data exclusions	8 of the individuals (6 control workers and 2 queens) that were originally sequenced were subsequently excluded, as inspection of the data suggested that these individuals were in fact members of the species Polistes gallicus, which is extremely phenotypically similar to Polistes dominula. All eight of these individuals belonged to the same two nests, and all exhibited greatly reduced rates of unique mapping to the P dominula genome relative to the other samples. Without a way to verify that these samples were the correct species, we erred on the side of caution and did not include the samples in any further analysis.<br><br>579 genes with low expression were excluded, as described in the methods.
Replication	Phenotypic data were measured twice by independent observers and were judged to be strongly replicable (Cohen's kappa > 0.9).
Randomization	Nests were assigned to treatment groups prior to the eclosion of workers using stratified random sampling.
Blinding	Blinding during the laboratory-based portion of the experiment was not possible, as the presence or absence of a queen on a nest is unavoidably obvious. All phenotypic measurements and RNA extractions were performed blind with respect to treatment group.

Reporting for specific materials, systems and methods

We require information from authors about some types of materials, experimental systems and methods used in many studies. Here, indicate whether each material, system or method listed is relevant to your study. If you are not sure if a list item applies to your research, read the appropriate section before selecting a response.

Materials & experimental systems

n/a	Involved in the study	
[x]	[ ]	Antibodies
[x]	[ ]	Eukaryotic cell lines
[x]	[ ]	Palaeontology and archaeology
[ ]	[x]	Animals and other organisms
[x]	[ ]	Human research participants
[x]	[ ]	Clinical data
[x]	[ ]	Dual use research of concern

Methods

n/a	Involved in the study	
[x]	[ ]	ChIP-seq
[x]	[ ]	Flow cytometry
[x]	[ ]	MRI-based neuroimaging

Animals and other organisms

Policy information about studies involving animals; ARRIVE guidelines recommended for reporting animal research

Laboratory animals	The study did not involve laboratory animals.
Wild animals	Polistes dominula paper wasps nests located near the Department of Biology, University of Florence were identified and observed daily until they reached a large enough size to be removed. Removals were performed early morning (while individuals were still subdued by the cool temperatures) by using a small pair of forceps to pull the nest off the wall or other surface by its petiole. Removed nests were immediately placed with all individuals into a jar and transported within ~30 minutes to the laboratory. A total of 146 foundress queens (female; approx age = 6 months) and 8 workers (female; approx age = 1 week) were transferred in this manner. At end of experiment, all samples were euthanised by decapitation using surgical scissors immediately followed by placement into ethanol (bodies) or RNAlater (heads).
Field-collected samples	Nests were housed in 30x30x30 cm glass boxes with wire mesh on top to allow inflow of fresh air. Water, nest materials and food (dipteran larvae) were provided ad libitum. Photoperiod was ambient (approx. 06:00-20:30); temperature was ambient on warm days, and supplemented by heaters on cool days (maximum temperature ~35C; minimum temperature ~15C). At end of experiment, individuals were removed early in the morning while temperatures remained cool and euthanised by decapitation using sterile surgical scissors, immediately followed by placement into ethanol (bodies) or RNAlater (heads).
Ethics oversight	Ethical oversight is not currently required for the use of arthropods for scientific purposes in the UK or in Italy.

Note that full information on the approval of the study protocol must also be provided in the manuscript.
