# Supplementary material for: The molecular basis of socially mediated phenotypic plasticity in a eusocial paper wasp
Source: Nat Commun. 2021 Feb 3;12:775. doi: 10.1038/s41467-021-21095-6 (PMC7859208; doi:10.1038/s41467-021-21095-6)
Supplement: Supplementary file 4 — Description of Additional Supplementary Files [file 41467_2021_21095_MOESM4_ESM.pdf]

Description of Additional Supplementary Files

Supplementary Data 1:

"Genes removed following each feature selection iteration of the support vector machine. RSMCVerror: The root mean squared 3-fold cross-validation error of the model that resulted following the removal of each given gene. Weight: The weight of each feature in the original, unoptimized SVM. Weights were calculated as the matrix product of the SVM's coefficients and its support vector values. Optimal: Whether the gene appears in the final, optimized caste-classifying SVM. DE: Whether the gene was identified by DESeq2 as differentially expressed between queens and workers."

Supplementary Data 2:

"Genes remaining in the final, optimized support vector machine. Weight: The weight of each feature in the original, unoptimized SVM. Weights were calculated as the matrix product of the SVM's coefficients and its support vector values."

Supplementary Data 3:

"Genes identified as differentially expressed between control workers and queens by DESeq2 at adjusted $p<0.05$. BaseMean: Mean expression of the gene in the reference group (queens). Log2LFC: Log(2) fold change of the gene in the comparison group (workers) compared to the reference group. LFCSE: Standard error of the log fold-change. Stat: Wald test statistic for the given comparison. pvalue: p-value for the given test statistic. padjusted: Adjusted p-values generated using Benjamini-Hochberg correction with alpha = 0.05."

Supplementary Data 4:

"Genes identified as differentially expressed between control workers and queen removal individuals by DESeq2 at adjusted $p<0.05$. BaseMean: Mean expression of the gene in the reference group (queens). Log2LFC: Log(2) fold change of the gene in the comparison group (workers) compared to the reference group. LFCSE: Standard error of the log fold-change. Stat: Wald test statistic for the given comparison. pvalue: p-value for the given test statistic. padjusted: Adjusted p-values generated using Benjamini-Hochberg correction with alpha = 0.05."

Supplementary Data 5:

"Genes identified as differentially expressed with scaled age among control workers by DESeq2 at adjusted $p<0.1$. BaseMean: Base mean expression (intercept) of the gene. Log2LFC: Log(2) fold change of the gene with a unit increase in age (as age was scaled, a unit increase here represents an increase of one standard deviation). LFCSE: Standard error of the log fold-change. Stat: Wald test statistic for the given comparison. pvalue: p-value for the given test statistic. padjusted: Adjusted p-values generated using Benjamini-Hochberg correction with alpha = 0.05."

Supplementary Data 6:

"Phenotypic data pertaining to samples analyzed in the study. Treatment: The treatment group to which the individual was assigned. C = sham removal; QR = queen removal; 3 = three-day observation following manipulation; 12 = twelve-day observation following

manipulation. Role: Whether individual was originally a foundress queen or a worker. Age: Age in days at time of sampling. Ovaries: Ovarian index at time of sampling, measured following Cini et al (2013). Elo: Elo rating for the individual based on intragroup dominance interactions in the three days prior to sampling, as described in Taylor et al (2020). Queenness: For queen removal individuals, caste identity at the time of sampling, measured as described in Taylor et al (2020). NestID: Nest from which the individual was collected."
