# Supplementary material for: The molecular basis of socially mediated phenotypic plasticity in a eusocial paper wasp
Source: Nat Commun. 2021 Feb 3;12:775. doi: 10.1038/s41467-021-21095-6 (PMC7859208; doi:10.1038/s41467-021-21095-6)
Supplement: Supplementary file 2 — Peer Review File [file 41467_2021_21095_MOESM2_ESM.pdf]

Reviewer #1 (Remarks to the Author):

Overview:

This manuscript, "The molecular basis of socially-mediated phenotypic plasticity in a eusocial paper wasp," examines the transcriptomes of a paper wasp as they relate to queen or worker status and the influence of queen removal on adult individuals that have the chance to transition to the role of a replacement reproductive queen. The phenotypic measurements of individual age, ovarian development, and behavioral dominance from this experiment were published previously – here the authors analyze transcriptomes from the brains of single individuals from the same experiment. The main conclusions of the study as I understand them from the abstract are (i) gene expression changes in the brains of individuals when the queen is removed, (ii) the transcriptional profiles of these individuals are intermediate to those of wild-type workers and queens, (iii) the transcriptomes exhibit changes in response to queen removal even when changes in ovarian development and dominance behaviors are not present, and thus (iv) large changes in gene expression can occur in the absence of change at the "phenotypic level," so transcriptomes are not "a proxy for the molecular underpinnings of the external phenotype."
The authors have generated an impressive data set with n=107 RNA-seq libraries from brains of individuals to correlate with phenotypic data from the same individuals. However, I had concerns about the confounding influence of age and the use of relatively abstract statistics in support of the story that behavioral and ovarian phenotypic variance is poorly reflected by brain transcriptomic variance.

Specific issues:

1. As I understand the SVM, the approach reduces the full set of genes down to those that are most differentiated in expression between wild-type queens and workers and then fits a model that produces a "queenness" metric based on the predictive power of collective transcription levels of these genes to identify queen or worker status. The authors then use this model to calculate a "queenness" score based on the transcription of these genes in workers that have had their queen removed, and test whether the transcriptional queenness metric is associated with a phenotypic queenness metric (based on ovarian development and dominance behaviors). My biggest concern about this experimental design is the extent to which the wild-type queens differ from workers in ways besides ovarian development and dominance – most notably, it is unclear if the queens were much older than all the other assayed individuals (i.e., previously overwintered spring foundresses of unknown but much older age). If this is the case some of the findings may be explained by confounding effects of the influence of ageing processes on SVM training. It seems notable that age appears to have a better model fit with the gene expression queenness metric (Fig. 2b) than the phenotypic SVM does (Fig. 2a). Is the training data set best capturing age-associated gene expression changes rather than reproductive status associated gene expression changes? And if so, does the queenness phenotype metric take into account all of the relevant aspects of physiological phenotypes required to support the main claims of the paper?

2. The same concerns extend to the WGCNA modules. Could more insight be gained by directly correlating expression levels of individual genes with trait measurements, rather than using modules of many genes that may be clustering (at least in part) according to aspects of ageing that are not related to reproductive status?

3. Box 1. It appears that very different sample sizes were used to train the phenotype SVM (queen n = 54, worker n = 220) versus the gene expression SVM (queen = 27, worker = 12). Is this correct? In particular, if 12 workers versus 220 workers were used this would seem to give a massive training advantage to the phenotypic SVM. Did the authors try making SVMs for each type of data using only the individuals with gene expression data?

4. L186: "the picture of caste differentiation provided by standard differential expression analysis appears to miss a great number of subtle differences in individuals' gene expression profiles that contribute to caste differentiation." Would the removal of the authors' criteria that differentially expressed genes must have a 1.5 fold difference be a simple way to catch these genes with subtle but significant differences in a differentially expressed gene (DEG) analysis? I also wonder if it is appropriate to directly compare SVM methods and DEG methods because they are measuring different things. The SVM summarizes what is happening across all the informative genes but cannot rank their effects, whereas DEG analysis summarizes what is happening with each individual gene and can be used to rank their effects. SVM cannot tell us was a DEG analysis does and vice versa.

5. Figure 1 would benefit from showing comparable phenotypic SVMs (as in Fig S1F, but with sequenced control individuals shown as well) in order to illustrate the different patterns between the SVM approaches (e.g., that workers that don't transition exhibit significant transcription differences from control workers but not significant phenotypic differences from control workers).

6. L68: "…significant progress has been made in unravelling the molecular underpinnings of… the reproductive castes of social insects" – I recommend the authors also cite the excellent study of adult reproductive and behavioral transitions in the ant Harpegnathos saltator, Gaspocic et al. 2017 Cell (I am not an author). This is an exceptional example of combining transcriptomic, phenotypic assays, and functional genetic screens. Harpegnathos is also likely a better biological analog to paper wasps than the clonal raider ants (in which all females lay eggs and there are no queens).

Reviewer #2 (Remarks to the Author):

In this paper Taylor et al., use RNA-seq analysis in brains of individual Polistes dominula workers to attempt to uncover the molecular basis of behavioural/phenotypic plasticity in this species. The use of individuals, rather than pools, makes this a very powerful and novel experiment. In addition, the authors use support vector machines to analyse their data, and the application of this technique which is novel for the field (although it is commonplace in analysis of cancer transcriptomes). Using this approach, they define gene expression signatures in the brains of workers after the loss of the queen, but this gene expression signature doesn't correspond to the workers' phenotype (as defined by ovarian development and dominance score). The authors interpret this as "transcriptomes are neither a direct reflection of the genotype nor a proxy for the molecular underpinnings of the external phenotype" (L43-44). This is a big claim and I think this conclusion is a bit overstated, especially given the lack of acknowledgement/discussion or evidence to either support or refute alternative conclusions (refer to point one below). I do, however, think that the data presented in this manuscript is robust and would be of interest to a range of researchers in sociobiology. Additionally, the manuscript is extremely well written and was a pleasure to read.

Major points

As alluded to above, the authors find that that there are substantial gene expression differences seen between workers from queen-removed colonies despite the fact that some of these individuals are phenotypically unchanged and the data doesn't indicate specific genes or processes that do underpin the enhanced ovarian development and dominance behaviours that were used as markers of phenotypic plasticity in this study (Taylor et al., 2020). The authors state, based on this data, that "transcriptomes are neither a direct reflection of the genotype nor a proxy for the molecular underpinnings of the external phenotype" (L43-44) and that this "calls into question the assumptions of the phenotypic gambit (L134-135). It is important to note that the authors' make this claim in the absence of any evidence/discussion for what IS responsible for the underlying phenotypic changes as workers transition into the queen-like reproductive role. I think that the manuscript would benefit from a wider discussion and consideration of alternative mechanisms and limitations of the current dataset that may affect the strength with which the authors' make this claim. In particular, how are

the differences in ovarian development and dominance modulated if not by gene expression? Given that, in some cases, there generally poor agreement between the transcriptome and proteome (e.g. Nuria Casas-Vila et al., Genome Res. July 2017 doi:10.1101/gr.213694.116), are the authors' suggesting regulation may be occurring at the post-transcriptional level in Polistes? Is it possible that plasticity is a two-stage process in Polistes with a priming event involving wide-spread gene expression changes followed by an activating event (that may require more small-scale expression changes)? Also, I am not a neurobiologist, but is it possible that the neurons required for dominance behaviour or indeed to control reproduction are only a small subset of the overall brain and that using the whole brain (and I realise there are very practical reasons for doing this) has swamped out the changes in gene expression that actually underlie these behaviours. Based on the points above I do feel that refuting the "phenotypic gambit" is an over-interpretation of their results and there is no direct evidence at this stage to support this. Note that I am not suggesting the authors do any further work, but rather that they widen their discussion regarding the lack of correlation between gene expression and phenotype and include a discussion of alternative explanations. Similarly, it would be good if the authors could discuss the limitations of phenotyping which may also help explain this lack of correlation. Although the phenotyping is not part of this manuscript it is highly relevant. Having read Taylor et al., 2020, the phenotyping does seem very robust – but was limited in nature to the two phenotypes discussed; ovarian morphology and dominance. Therefore, is it possible that the gene expression signatures discovered in this study may be associated with other, unmeasured, phenotypes?

I was intrigued with the finding that age is the biggest factor that drives differences in gene expression in this study. Do the authors have age data for the worker (in presence of queen) RNA-seq that was used to train the SVM? If so, could this data be used to separate the pure effects of aging on brain gene expression from those that are associated specifically with the caste transition? Also, how does this relate to the natural history of Polistes? If older workers leave the nest to found their own nest, then perhaps this age-related plasticity is more important (in an evolutionary sense) than the plasticity to detect the queen-loss. My concern is that if age is the biggest determinant of caste in Polistes then the authors aren't examining plasticity specifically. Is it possible to differentiate between plastic genes vs. age-related genes? Perhaps comparing 'old' workers with low and high changes in phenotype vs. 'young' workers would achieve this? It is not clear whether this is possible with the current data set but it would be good for the authors to discuss the relevance of age being a driving factor in establishing gene expression patterns and what this means for the understanding of plasticity in this species in a bit more depth.

This manuscript has generated a wealth of gene expression data, but the discussion of this data in detail is somewhat limited (L275-292). Is there any overlap in differentially expressed genes with those that are known to control reproduction or dominance/aggression in other species e.g. Drosophila? Do these data hint at the biological processes that are underpinning the transition from worker to queen?

Minor points:
L67 "controlling for genetic differences". I know what the author's mean here – that all workers are totipotent irrespective of genetic differences, but could the authors' reword this to reflect the fact the queens are genetically distinct and, although nestmates are related they are not genetically identical. Also, are P. dominula monandrous or polyandrous?

L109 Brain or head? If whole heads were used there will be contributions in the RNA- expression profile of non, brain tissues and this should be discussed explicitly.

Fig. 1 – I am not familiar with SVM analysis – what is the "classifier estimate" a measure of?

L183 – not clear which "four" the authors are referring to.

L273 what is the rationale for these modules being “of particular interest”

Reviewer #3 (Remarks to the Author):

Review of « The molecular basis of socially-mediated phenotypic plasticity in a eusocial paper wasp” by Taylor et al.

In this study, the authors use a novel technic using machine learning to understand the link between changes in phenotypes and gene expression profiles. In particular, the authors aim to address the changes in gene expression following the loss of a queen in a social insect organism, that will lead to some workers undergoing significant phenotypic changes to replace the queen. They demonstrate that important gene expression changes may occur even if phenotypic changes cannot be perceived.

I find the manuscript interesting and the question it addresses relevant for our field, the machine learning analysis is a fascinating technical approach. However, a lot of technical details regarding this approach are left out of the manuscript which leaves the reader wondering about the results. The manuscript also lacks detailed methods that would make the work difficult to reproduce as such.
As a result, I have some major comments that I would like the authors to address before seeing this work published.

Major comments
My main concern is about the methods description. As the authors mention in the introduction, this is a novel technique in the field of insect social research and not all readers will understand how this machine learning approach function, I actually don’t, just by reading the methods, so I am unable to access whether the procedure and the parameters are correctly applied. More background on the methodology could help understand the rationale behind it.

The authors correctly explain how useful machine learning may be (even though the introduction just very shortly mentions it) but fail to really explain how this all works. As a result, I am not sure I really truly understand how the sets of genes found via machine learning is associated with queens or worker expression profiles. What about their expression patterns? Are all of them associated with the castes, associated with both queen and worker profiles, or some with just one caste? It might be easier to grasp how these genes are in fact associated with the caste profiles if we could visualize their expression profiles. Are there any patterns of caste differentiation in these genes (even if not significant)? On which criteria are these genes selected by the SVM approach?

Then the authors used this set of genes (if I understood correctly, perhaps I did not), and look for changes in their expression profiles when the queen is removed from the colony, while trying to link the changes at the gene level with phenotypic changes. What is a SVM estimate? (L- 209), how do you conclude that some individuals have expression profiles intermediate between queens and workers? This part is very unclear to me. It is obvious on a PCA plot, but I found hard to understand the result section and especially the SVM part.

The authors also state that because they could not perceive any visible/striking phenotypic changes when queens were removed for the majority of the workers, it was surprising to see that a majority of workers had an intermediate expression profile. But could these gene expression changes be related to some more basic physiological changes that may not be seen such as hormonal responses?

My second concern is that this manuscript relies heavily on previous results already published, which in itself is not an issue. However, the method section of this manuscript omits a lot of information, such as how many samples for each group were used for the analysis, how/when/where the samples

were collected. In this regard, we need to read the first study to understand the study design, instead a small brief paragraph summarizing this would be useful.

For instance, I could not find any information on the queens and workers used for transcriptomic analysis. Age? Was the study done without regard to the complexities of these variables?

Authors used brain data for gene expression, the specific reasoning behind using this particular tissue compared to more queen/worker associated tissues such as ovaries is not explained in the manuscript. Would you expect to find such a high number of genes associated with caste profiles if you were using different tissues?

My one stylistic suggestion is that part of this paper is very 'technical' in that the data are heavily processed for the support vector machine classification and the results concerning this analysis are a bit opaque. Thus, I would urge the authors to extend themselves a bit and try to make their results a bit clearer to the casual reader. For instance, the methods are not explained very clearly L430. What are classifiers? What is parameter space?

Lastly, we end up with, yet another set of genes associated with castes. Besides the GO terms, perhaps it would be good to see whether this set of 2000 genes found through machine learning actually match what is already known from previous caste gene expression studies who have looked closely at caste-specific gene expression in your study organism.

Minor comments

Introduction

I find the introduction well written overall, perhaps more examples of machine learning studies and how they are applied to transcriptomic/behavioral data would enable the readers to completely adhere to your methods.

L 122. “given their sensitivity” what do the authors mean there? Sensitive to changes in expression? How so?

Methods

L. 426 – The filtering threshold seems a bit arbitrary, what does 20 counts represent in terms of cpm? Is it a threshold that is recommended for Support Vector machine classification?

L. 453 – why using such stringent cut-off? Are there any differences in the number of DEGs when omitting this threshold and relying exclusively on pvalue?

L. 460 – WGCNA has an in-house filtering option that removed genes with too few values. Why did you decide to use your own filtering threshold? Or is this 10% threshold based on actual WGCNA analysis? Do the two approaches differ in the number of genes left after filtering? I recognize that WGCNA recommend a 10% threshold minimum, however the threshold used in a study should be based on experimental design, sequencing depth and sample counts.

L 463 – Would it be possible to add some figures of the WGCNA methods? Such as soft-threshold power figure and the eigengene correlation figure to merge the modules?

Also, were the data transformed before being used for WGCNA analysis as recommended by the authors?

L. 469 – For the GO terms analysis, is the genome used in this study annotated? What about genes that do not have orthologues with D. melanogaster? That could lead to a bias in your results. How many genes were assigned orthologs? Doesn't NCBI provide quite a comprehensive annotation for every published genome? Why not using this?

Results

L. 163 – is 25% a good value? Without background on the technique it is very difficult for the reader to make up its mind about this result (if this is even important).

L. 180 – Perhaps the low number of DEGs is also due to your cut off at LogFC 1.5 – how many more genes do you get when you include all genes with a significant transformed pvalue? Do they also belong to your list of 1992 genes from the SVM analysis?

L. 185 – You are basically testing only one method. If you would run the DEG analysis with a different software you might get a bigger data set, and perhaps those genes would not be included in your final SVM dataset. To me it is hard to judge whether your SVM technic really captures almost all the information.

L. 194 – is that a method section?

L. 200 – Were the nest used as a random factor in the DEG analysis?

L. 208 – The following paragraphs could be moved to the discussion, at least part of it sound very much like a discussion

L 326. Once again, I do not understand what classifiers are, and what their variation really means, so the figure is quite obscure to me.

L. 275 – The WGCNA analysis. Were genes included in your three modules of interest also part of your 1922 genes from the SVM analysis? Is WGCNA a comprehensive approach to capture genes involved with the caste specific gene expression profiles and following perturbation?

Discussion

The authors found quite a large set of genes (around 20% of the total amount of genes in the genome) that were associated with the caste profiles using machine learning. We do not however understand how these genes are "associated", are they associated with one of the castes? With their behavior/phenotypic traits?

What about the WGCNA results? How do they fit with your hypothesis and conclusions? I cannot see a mention of them in the discussion

Reviewer #4 (Remarks to the Author):

The manuscript "The molecular basis of socially-mediated phenotypic plasticity in a eusocial paper wasp" explores how brain gene expression shifts in adult wasps as they transition from a queenright (where all workers are subordinate) to a queenless (where workers compete for reproductive dominance) colony. The authors use a unique method to classify individuals on a one-dimensional linear scale ("queenness") based on behavioural (dominance index) and physiological (ovarian index) data. The results show that the 'queenness' index, as well as age can be used to explain the shift and variation in brain gene expression among queenless workers compared to queenright workers. Moreover, the authors highlight three modules of genes (and GO terms) that are co-expressed and correlate with queenness among workers in queenless colonies. The novelty and the broadness of scope are clearly outlined in the introduction and discussion. Moreover, by using Bayesian logistic regression model and SVM approach, the authors took complex behavioural, physiological, and genomic data, and simplified them to compare caste (queenness, Bayesian) and gene expression (SVM classifying module). The results were clearly presented, and conclusions drawn from those results were clear. Overall, the methods and the results can be built upon in future studies that explore how behavioural and physiological shifts may correspond to changes in gene expression - in a variety of animal systems, including, but not limited to, other species of social insects.

I only have a few minor comments that the authors may want to consider.
Line 59 & 376: Should this term be "bases" or "basis"?

Line 108: Consider removing "To do so," as the clause it is referring to is in the previous paragraph.

Line 109: The methods make it clear that the whole brain transcriptome is being sequenced, but here "bran gene expression profiles" is used. It wasn't always clear that this was the whole transcriptome until line 112 (even though it's alluded to in line 87 as well). I think somehow this could be clarified (perhaps line 109 "assaying the transcriptome of the brain...").

Line 159: Similar to the point above "We trained an SVM using gene expression data..." It might be clearer if it said "whole transcriptome data" (to highlight the scope of the project).
Figure 1 tells an important part of the narrative, that overall brain gene expression shifted after queen removal. However, after reading about the "queenness" index, it seemed a bit odd that this was not a figure with the x-axis as "queenness" and the y-axis as the classifier estimate (as is shown in Fig 2).

Figure 2 was a bit complicated. Perhaps A-C could be combined with Figure 1 instead?
- Check colour codes in A-C. Blue now represents something different than in Fig 1. The colour codes also shifted in the Supplemental Figure.
- Y-axis is SVM Classifier Index (this took a minute to figure out from the caption)
- Is there a reason why Age v Queenness figure is not shown? Perhaps as an inset? The relationship is alluded to in the discussion as well (lines 308-9).
- D-F: Why are there colours used to name the modules? It might be easier to interpret if they were just numbers or letters. Also - consider placing the column titles on top, and labeling the first column (with the numbers:colours ID) as "Module". I didn't notice it as a title on the left.
- G-H: please define BP, CC, and MF in the figure caption.

Lines 275-292: The order in which these modules are presented is awkward - particularly since 11 and 8 are highlighted in Figure 2, and 2 is discussed in the middle of the paragraph.

Response to referees: The molecular basis of socially-mediated phenotypic plasticity in a eusocial paper wasp (Ref: NCOMMS-20-28189A)

We would like to thank the four anonymous reviewers for their detailed and insightful comments. We have considered the comments and detail our responses and modifications to the manuscript below. Line numbers refer to the copy of the revised manuscript with tracked changes.

General changes

Four concerns arose repeatedly across multiple reviewers' comments. We address these collectively here, rather than writing duplicate responses to each relevant comment.

1. A possible confounding effect of age on SVM classifications

Three reviewers noted that, since foundress queens differ from early season workers not only in their caste but also in their age, it is possible that age is a confounding variable in our SVM classifier (Comment 1 by Reviewer 1; Comment 3 by Reviewer 2; Comment 3 by Reviewer 3). While it is clear from our analyses that age is a major determinant of expression profiles, we believe that we can rule out the possibility that our queen–worker classification is dominated by these effects on three grounds. First, if age is an important factor in our model, then control workers and queen removal workers should not differ strongly in their SVM classifications (since they represent contemporary cohorts and queen removal has no effect on individuals' ages). Contrary to this expectation, there are no clear differences in the classification scores of these groups. Second, variation in the classifier estimates generated for control workers does not correlate with age, as would be the case if our training data were primarily separated by age. Third, using DESeq2 we have identified a set of several hundred genes whose expression correlates with age among control workers: this set of genes does not overlap significantly with the set identified by feature selection in our paper. Taken together, we feel that these three results are sufficient to show that our SVM model is properly classifying individuals by caste identity rather than age. We have added a paragraph to the results to this effect (lines **324-336**).

2. Effects of log fold change thresholds on DESeq2 results

Two reviewers noted that the disparity in the number of genes we identified using DESeq2 and SVM feature selection might be driven by the log fold change threshold we had applied as part of our DESeq2 approach (Comment 4 by Reviewer 1; Comments 10-11 by Reviewer 3). We chose to apply such a threshold to be in line with common practice in our field (e.g. Chalfin

et al 2014; Gacias et al 2016) and others (e.g. Makhijani et al 2018; Staudt et al 2018), but we acknowledge that doing so creates a somewhat artificial difference between the traditional gene-based approach and the SVM. We have therefore updated the analyses of differential expression with new results that do not apply a fold change threshold (lines **224-231**). As expected, omission of this threshold increases the number of genes to over 2000 that show differential expression according to DESeq2. Notably, this set of genes exhibits only a partial overlap with the set identified by SVM feature selection, and an SVM model trained using the full DESeq2-identified gene set had a significantly greater error rate than the feature selection-optimised model (lines **229-231**). This suggests that our feature selection process is still more sensitive in identifying genuinely caste-predictive expression patterns than an approach based on differential expression of individual genes. We have amended the methods to reflect our change in approach (lines **686-690**) and have rewritten part of the discussion to assess the difference of approaches (lines **529-560**), but have removed the passages that suggested that our approach identifies expression patterns reflecting caste in a strictly larger set of genes than the traditional differential expression analysis.

3. Insufficient clarity of SVM descriptions

Two reviewers considered the explanation of either the SVM process itself or the correct interpretation of the SVM classifications too brief and insufficient to provide a good understanding of the approach (Comment 8 by Reviewer 2; Comments 1, 5 and 21 by Reviewer 3). We have expanded the methods section to outline the SVM methodology in more depth, which we hope will make our methods accessible, and transferable for readers who wish to apply SVM classification to their own data (lines **657-672**). We have also more explicitly explained the basic methodology by which we applied SVM classification to our data in the results section, which we hope will ensure that readers fully understand the context for the results (lines **160-173; 184-193**). We have especially focused on making clear the correct interpretation of the term 'SVM classification', which was repeatedly flagged to us as a source of confusion by the reviewers (lines **166-170; 204-206**).

4. Insufficient discussion of alternative explanations

Two reviewers felt that we had overstated our case when we claimed in the paper that our results reveal a disconnect between gene expression and phenotypic expression (Comment 1 by Reviewer 1; Comments 1-3 by Reviewer 2). In the light of these comments, we have edited several portions of the abstract, introduction and discussion to present a more measured claim on the relationships (or lack therefore) between gene expression and caste phenotype (lines **40-46; 133-134; 138-139; 414; 425-430**). We have also added a section to the discussion in which we explicitly discuss the limited number of phenotypes measured (for example, the fact that we did not assess hormone levels; lines **432-448**).

Reviewer comments
(*reviewer comments are in italics*; author responses are in roman case)

Reviewer 1:

Overview:

This manuscript, "The molecular basis of socially-mediated phenotypic plasticity in a eusocial paper wasp," examines the transcriptomes of a paper wasp as they relate to queen or worker status and the influence of queen removal on adult individuals that have the chance to transition to the role of a replacement reproductive queen. The phenotypic measurements of individual age, ovarian development, and behavioral dominance from this experiment were published previously – here the authors analyze transcriptomes from the brains of single individuals from the same experiment. The main conclusions of the study as I understand them from the abstract are (i) gene expression changes in the brains of individuals when the queen is removed, (ii) the transcriptional profiles of these individuals are intermediate to those of wild-type workers and queens, (iii) the transcriptomes exhibit changes in response to queen removal even when changes in ovarian development and dominance behaviors are not present, and thus (iv) large changes in gene expression can occur in the absence of change at the "phenotypic level," so transcriptomes are not "a proxy for the molecular underpinnings of the external phenotype."

The authors have generated an impressive data set with n=107 RNA-seq libraries from brains of individuals to correlate with phenotypic data from the same individuals. However, I had concerns about the confounding influence of age and the use of relatively abstract statistics in support of the story that behavioral and ovarian phenotypic variance is poorly reflected by brain transcriptomic variance.

Specific issues:

1. As I understand the SVM, the approach reduces the full set of genes down to those that are most differentiated in expression between wild-type queens and workers and then fits a model that produces a "queenness" metric based on the predictive power of collective transcription levels of these genes to identify queen or worker status. The authors then use this model to calculate a "queenness" score based on the transcription of these genes in workers that have had their queen removed, and test whether the transcriptional queenness metric is associated with a phenotypic queenness metric (based on ovarian development and dominance behaviors). My biggest concern about this experimental design is the extent to which the wild-type queens differ from workers in ways besides ovarian development and dominance – most notably, it is unclear if the queens were much older than all the other assayed individuals (i.e.,

previously overwintered spring foundresses of unknown but much older age). If this is the case some of the findings may be explained by confounding effects of the influence of ageing processes on SVM training. It seems notable that age appears to have a better model fit with the gene expression queenness metric (Fig. 2b) than the phenotypic SVM does (Fig. 2a). Is the training data set best capturing age-associated gene expression changes rather than reproductive status associated gene expression changes? And if so, does the queenness phenotype metric take into account all of the relevant aspects of physiological phenotypes required to support the main claims of the paper?

We thank the reviewer for this comment regarding the possibility that age rather than caste per se is the primary driver of our SVM results, which we have addressed with a number of additional analyses (**General Changes, Section 1**; lines **324-336**). In response to the reviewer's observation that there may be relevant phenotypic aspects that we have failed to take into account in the study, we agree that this is a possibility that we cannot rule out. We have added a section to the discussion to acknowledge the potential limitations of our phenotyping, while also clarifying our belief that behaviour and reproductive development are the most important and representative aspects of caste expression (lines **432-448**).

2. The same concerns extend to the WGCNA modules. Could more insight be gained by directly correlating expression levels of individual genes with trait measurements, rather than using modules of many genes that may be clustering (at least in part) according to aspects of ageing that are not related to reproductive status?

Using WGCNA modules allows us to relate the (correlated) expression of all genes in a module with individual phenotypic traits, including age. Assessing genes individually would allow us to produce equivalent results, but would not be more insightful regarding the association of expression with phenotypes, nor have allowed us to assess the broader patterns of gene loss that accompany queen succession that are the focus of our study. We have, however, added a more nuanced measure of the relationship between gene modules and phenotypic identity, in which we assess the degree to which the significance of genes within a module (their connectivity within that module) is correlated with the predictive power of those genes with regards to phenotypic queenness (lines **375-378**). We feel that the presence of a strong correlation between these two factors (as occurs in Module 11) strongly indicates that the association between the module and the trait is more than incidental.

3. Box 1. It appears that very different sample sizes were used to train the phenotype SVM (queen n = 54, worker n = 220) versus the gene expression SVM (queen = 27, worker = 12). Is this correct? In particular, if 12 workers versus 220 workers were used this would seem to give

a massive training advantage to the phenotypic SVM. Did the authors try making SVMs for each type of data using only the individuals with gene expression data?

This is a good suggestion. We have retrained the Bayesian logistic regression model that we used to estimate phenotypic caste identity (queenness) using just the samples that were sequenced. Regressing the queenness estimates thus produced against age and SVM estimates produces near-identical results to those in the original submission, which suggests that the training advantage has not qualitatively affected our results. We have updated the results section to incorporate this result (lines **306-308**).

4. L186: "the picture of caste differentiation provided by standard differential expression analysis appears to miss a great number of subtle differences in individuals' gene expression profiles that contribute to caste differentiation." Would the removal of the authors' criteria that differentially expressed genes must have a 1.5 fold difference be a simple way to catch these genes with subtle but significant differences in a differentially expressed gene (DEG) analysis?.

We have made a number of modifications to the manuscript to address the possible influence of the fold change threshold we applied to our DESeq2 analysis (**General Changes, Section 2**; lines **224-231; 529-560**).

I also wonder if it is appropriate to directly compare SVM methods and DEG methods because they are measuring different things. The SVM summarizes what is happening across all the informative genes but cannot rank their effects, whereas DEG analysis summarizes what is happening with each individual gene and can be used to rank their effects. SVM cannot tell us was a DEG analysis does and vice versa

We have added substantially to the discussion to more strongly emphasise that the SVM analysis represents a complement (rather than an alternative) to standard approaches (lines **526-560**).

5. Figure 1 would benefit from showing comparable phenotypic SVMs (as in Fig S1F, but with sequenced control individuals shown as well) in order to illustrate the different patterns between the SVM approaches (e.g., that workers that don't transition exhibit significant transcription differences from control workers but not significant phenotypic differences from control workers).

As suggested, we have altered **Figure 1** so that it now displays measures of both phenotypic queenness and SVM classifications.

6. L68: "…significant progress has been made in unravelling the molecular underpinnings of… the reproductive castes of social insects" – I recommend the authors also cite the excellent study of adult reproductive and behavioral transitions in the ant Harpegnathos saltator, Gaspocic et al. 2017 Cell (I am not an author). This is an exceptional example of combining transcriptomic, phenotypic assays, and functional genetic screens. Harpegnathos is also likely a better biological analog to paper wasps than the clonal raider ants (in which all females lay eggs and there are no queens).

Thanks to the reviewer highlighting this very interesting paper, which we have now cited (line **73**).

<u>Reviewer 2:</u>

In this paper Taylor et al., use RNA-seq analysis in brains of individual Polistes dominula workers to attempt to uncover the molecular basis of behavioural/phenotypic plasticity in this species. The use of individuals, rather than pools, makes this a very powerful and novel experiment. In addition, the authors use support vector machines to analyse their data, and the application of this technique which is novel for the field (although it is commonplace in analysis of cancer transcriptomes). Using this approach, they define gene expression signatures in the brains of workers after the loss of the queen, but this gene expression signature doesn't correspond to the workers' phenotype (as defined by ovarian development and dominance score). The authors interpret this as "transcriptomes are neither a direct reflection of the genotype nor a proxy for the molecular underpinnings of the external phenotype" (L43-44). This is a big claim and I think this conclusion is a bit overstated, especially given the lack of acknowledgement/discussion or evidence to either support or refute alternative conclusions (refer to point one below). I do, however, think that the data presented in this manuscript is robust and would be of interest to a range of researchers in sociobiology. Additionally, the manuscript is extremely well written and was a pleasure to read.

Major points

1. As alluded to above, the authors find that that there are substantial gene expression differences seen between workers from queen-removed colonies despite the fact that some of these individuals are phenotypically unchanged and the data doesn't indicate specific genes or processes that do underpin the enhanced ovarian development and dominance behaviours

that were used as markers of phenotypic plasticity in this study (Taylor et al., 2020). The authors state, based on this data, that "transcriptomes are neither a direct reflection of the genotype nor a proxy for the molecular underpinnings of the external phenotype" (L43-44) and that this "calls into question the assumptions of the phenotypic gambit (L134-135). It is important to note that the authors' make this claim in the absence of any evidence/discussion for what IS responsible for the underlying phenotypic changes as workers transition into the queen-like reproductive role. I think that the manuscript would benefit from a wider discussion and consideration of alternative mechanisms and limitations of the current dataset that may affect the strength with which the authors' make this claim. In particular, how are the differences in ovarian development and dominance modulated if not by gene expression?

We agree with the reviewer's assessment that we have overstated the conclusiveness of our results with respect to the disconnect between gene expression and phenotypic expression, and we have rewritten several sections of the manuscript to better reflect the limitations of this approach (**General Changes, Section 4**; lines **40-46; 133-134; 138-139; 414; 432-461**).

2. Given that, in some cases, there generally poor agreement between the transcriptome and proteome (e.g. Nuria Casas-Vila et al., Genome Res. July 2017 doi:10.1101/gr.213694.116), are the authors' suggesting regulation may be occurring at the post-transcriptional level in Polistes? Is it possible that plasticity is a two-stage process in Polistes with a priming event involving wide-spread gene expression changes followed by an activating event (that may require more small-scale expression changes)? Also, I am not a neurobiologist, but is it possible that the neurons required for dominance behaviour or indeed to control reproduction are only a small subset of the overall brain and that using the whole brain (and I realise there are very practical reasons for doing this) has swamped out the changes in gene expression that actually underlie these behaviours. Based on the points above I do feel that refuting the "phenotypic gambit" is an over-interpretation of their results and there is no direct evidence at this stage to support this. Note that I am not suggesting the authors do any further work, but rather that they widen their discussion regarding the lack of correlation between gene expression and phenotype and include a discussion of alternative explanations.

We would like to thank the reviewer for these insightful comments. The correlation between the transcriptome and proteome is beyond the scope of this study, but would be an interesting future line of research, especially as the proteome is thought to be the closest molecular representation of the phenotype (Valcu and Kempenaers 2014; Bengston et al 2018). We were also intrigued by the suggestion that our results could reflect some kind of 'priming' of individuals' gene expression, which is not a process that we had previously considered, and fits well with our hypothesis that the post-removal changes might reflect adaptation to multiple successive instances of queen loss. We have incorporated this idea

into the section of our discussion in which we speculate as to the causes of the changes observed in post-removal individuals (lines **488-492**).

Regarding the reviewer's comments about our choice of tissue, we do agree that this is a topic that requires acknowledgement within the manuscript and we have edited the discussion to explicitly address our choice to sequence whole brain tissue rather than individual subdivisions of this tissue (lines **454-461**). We have also reworked the manuscript to temper our previous claim that our results undermine the phenotypic gambit (**General Changes, Section 4**; lines **138-139**).

3. Similarly, it would be good if the authors could discuss the limitations of phenotyping which may also help explain this lack of correlation. Although the phenotyping is not part of this manuscript it is highly relevant. Having read Taylor et al., 2020, the phenotyping does seem very robust – but was limited in nature to the two phenotypes discussed; ovarian morphology and dominance. Therefore, is it possible that the gene expression signatures discovered in this study may be associated with other, unmeasured, phenotypes?

We now explicitly discuss the limitations of our phenotyping approach (lines **432-461**).

3. I was intrigued with the finding that age is the biggest factor that drives differences in gene expression in this study. Do the authors have age data for the worker (in presence of queen) RNA-seq that was used to train the SVM? If so, could this data be used to separate the pure effects of aging on brain gene expression from those that are associated specifically with the caste transition?

As described in **General Changes, Section 2**, we have attempted to address the possibility that age is responsible for our SVM classifications by showing that age does not predict variation among control workers (lines **331-332**) and that caste-informative genes do not overlap significantly with age-responsive genes (lines **332-334**).

4. Also, how does this relate to the natural history of Polistes? If older workers leave the nest to found their own nest, then perhaps this age-related plasticity is more important (in an evolutionary sense) than the plasticity to detect the queen-loss. My concern is that if age is the biggest determinant of caste in Polistes then the authors aren't examining plasticity specifically. Is it possible to differentiate between plastic genes vs. age-related genes? Perhaps comparing 'old' workers with low and high changes in phenotype vs. 'young' workers would achieve this? It is not clear whether this is possible with the current data set but it would be good for the authors to discuss the relevance of age being a driving factor in establishing gene

expression patterns and what this means for the understanding of plasticity in this species in a bit more depth.

Generally speaking, age is not thought to be an important determinant of caste in *Polistes* and workers will rarely (if ever) leave the nest to found a new one in a temperate species like P. dominula. Moreover, it is generally expected that wasps emerging later in the season in simple societies such as those of *Polistes* will be larger and healthier, because the efficiency of nests' foraging and brood care activities increases as the nest grows in size. Indeed, in temperate species gynes (who will go on to be the next year's foundress queens) are generally both the latest-emerging and therefore 'youngest' individuals and also the largest. Nonetheless, we have performed additional analyses to try to disentangle the effects of age and caste in our analyses as described in **General Changes, Section 1**, showing that age-responsive genes do not appear to overlap with caste-informative genes identified by the SVM (lines **332-334**).

5. This manuscript has generated a wealth of gene expression data, but the discussion of this data in detail is somewhat limited (L275-292). Is there any overlap in differentially expressed genes with those that are known to control reproduction or dominance/aggression in other species e.g. Drosophila? Do these data hint at the biological processes that are underpinning the transition from worker to queen?

While we were unable to locate papers that list candidate genes for reproduction or aggression in *Drosophila*, we have compared our list of caste-biased genes to a set of genes that correlate with vitellogenin (which is itself known to correlate with aggression in this species; Manfredini et al 2018) in another *P. dominula* paper, Standage et al (2016; lines **497-501**). We have also used caste differential expression data from that paper to contextualise our seemingly surprising finding that GO terms for aggression and reproduction are not among the enriched terms identified by our own study (lines **501-512**), and we have expanded the section of the discussion in which we speculate about the possible role of epigenetic processes as drivers of phenotypic plasticity in our experiment (lines **484-492**). We hope that these additions have resulted in a more satisfying discussion section, which we acknowledge may have previously been unduly focused on methological details.

Minor points:

6. L67 "controlling for genetic differences". I know what the author's mean here – that all workers are totipotent irrespective of genetic differences, but could the authors' reword this

to reflect the fact the queens are genetically distinct and, although nestmates are related they are not genetically identical. Also, are P. dominula monandrous or polyandrous?

Thank you, in hindsight we realise that this wording is indeed rather misleading. We have rephrased to make our meaning clearer (lines **68-70**). To the reviewer's second point, *Polistes* wasps are almost exclusively monandrous, although rare cases of polyandry have been recorded. As we did not genotype our samples, we have conservatively chosen not to address the question of within-group relatedness, but our expectation is that all individuals from a nest should be full sibs.

7. L109 Brain or head? If whole heads were used there will be contributions in the RNA-expression profile of non, brain tissues and this should be discussed explicitly.

Exclusively brain tissue was used, as stated in the methods (line **637**). We have amended the discussion to address our choice of tissue, which should reduce any ambiguity on this point (lines **432-461**).

8. Fig. 1 – I am not familiar with SVM analysis – what is the "classifier estimate" a measure of?

We have amended the results section and the caption to Figure 1 to make it clearer that this term refers to the SVM-estimated transcriptomic similarity of individuals to control workers and queens (lines **166-170; 204-206**).

9. L183 – not clear which "four" the authors are referring to.

We have reworded this line for clarity (lines **213-214**).

10. L273 what is the rationale for these modules being "of particular interest"

These modules were notable for being significantly correlated with phenotypic traits in at least one focal group. We have rephrased the line in question to make this clearer (lines **352-354**).

<u>Reviewer 3:</u>

Review of « The molecular basis of socially-mediated phenotypic plasticity in a eusocial paper wasp" by Taylor et al.

In this study, the authors use a novel technic using machine learning to understand the link between changes in phenotypes and gene expression profiles. In particular, the authors aim to address the changes in gene expression following the loss of a queen in a social insect organism, that will lead to some workers undergoing significant phenotypic changes to replace the queen. They demonstrate that important gene expression changes may occur even if phenotypic changes cannot be perceived.

I find the manuscript interesting and the question it addresses relevant for our field, the machine learning analysis is a fascinating technical approach. However, a lot of technical details regarding this approach are left out of the manuscript which leaves the reader wondering about the results. The manuscript also lacks detailed methods that would make the work difficult to reproduce as such. As a result, I have some major comments that I would like the authors to address before seeing this work published.

Major comments

1. My main concern is about the methods description. As the authors mention in the introduction, this is a novel technique in the field of insect social research and not all readers will understand how this machine learning approach function, I actually don't, just by reading the methods, so I am unable to access whether the procedure and the parameters are correctly applied. More background on the methodology could help understand the rationale behind it. The authors correctly explain how useful machine learning may be (even though the introduction just very shortly mentions it) but fail to really explain how this all works. As a result, I am not sure I really truly understand how the sets of genes found via machine learning is associated with queens or worker expression profiles. What about their expression patterns? Are all of them associated with the castes, associated with both queen and worker profiles, or some with just one caste? It might be easier to grasp how these genes are in fact associated with the caste profiles if we could visualize their expression profiles. Are there any patterns of caste differentiation in these genes (even if not significant)? On which criteria are these genes selected by the SVM approach?

From the reviewer's comments, it is clear that we not adequately explained the SVM and feature selection processes. We have expanded the methods section to give a more explicit description of the SVM procedure (lines **657-672**), and we have additionally edited our results section to make it clearer that the SVM functions in a manner that is essentially similar to multivariate linear regression (lines **156-162**). We have also attempted to reinforce the fact that support vector classifications represent individuals' gene expression similarity to control

castes throughout (lines **166-170**; **204-206**). Finally, we have amended our description of the feature selection process, which we hope will make it clearer that this was the process by which an optimally caste-predictive set of genes was identified (lines **184-193**).

Then the authors used this set of genes (if I understood correctly, perhaps I did not), and look for changes in their expression profiles when the queen is removed from the colony, while trying to link the changes at the gene level with phenotypic changes. What is a SVM estimate? (L- 209), how do you conclude that some individuals have expression profiles intermediate between queens and workers? This part is very unclear to me. It is obvious on a PCA plot, but I found hard to understand the result section and especially the SVM part.

We have amended the manuscript to reinforce the fact that support vector classifications represent individuals' gene expression similarity to control castes (lines **166-170**; **204-206**). We hope that the edits made to **Figure 1**, directly juxtaposing the phenotypic logistic regression classifications with the transcriptomic SVM classifications, will also make this point clearer. The manner in which our SVM approach classifies individuals' gene expression profiles can be considered quite strongly analogous to the manner in which a multivariate regression model classifies samples' phenotypic profiles, and as the latter methodology is widely understood, we hope that making this parallel explicit in the figure and text (line **160-163**) will help make our methodology more easily intelligible to non-specialists.

The authors also state that because they could not perceive any visible/striking phenotypic changes when queens were removed for the majority of the workers, it was surprising to see that a majority of workers had an intermediate expression profile. But could these gene expression changes be related to some more basic physiological changes that may not be seen such as hormonal responses?

We have added a section to the discussion to explicitly address the limitations of our phenotyping (lines **432-448**).

2. My second concern is that this manuscript relies heavily on previous results already published, which in itself is not an issue. However, the method section of this manuscript omits a lot of information, such as how many samples for each group were used for the analysis, how/when/where the samples were collected. In this regard, we need to read the first study to understand the study design, instead a small brief paragraph summarizing this would be useful.

We have added a paragraph to the beginning of the methods section to provide this missing information (lines **615-633**), which we thank the reviewer for bringing to our attention.

3. For instance, I could not find any information on the queens and workers used for transcriptomic analysis. Age? Was the study done without regard to the complexities of these variables?

Data regarding the age, ovarian development, etc of sequenced individuals are available in the supplementary materials (**Table S7**). We have amended the methods to make our sampling methodology clearer (lines **629-633**)—thank you for making us aware that this was not explained previously.

4. Authors used brain data for gene expression, the specific reasoning behind using this particular tissue compared to more queen/worker associated tissues such as ovaries is not explained in the manuscript. Would you expect to find such a high number of genes associated with caste profiles if you were using different tissues?

We have amended the methods section to address and explain our choice of tissue, which we agree might well have been impactful upon our results (lines **432-461**).

5. My one stylistic suggestion is that part of this paper is very 'technical' in that the data are heavily processed for the support vector machine classification and the results concerning this analysis are a bit opaque. Thus, I would urge the authors to extend themselves a bit and try to make their results a bit clearer to the casual reader. For instance, the methods are not explained very clearly L430. What are classifiers? What is parameter space?

We have amended several portions of the results to more clearly explain our usage of SVM classification (**General Changes, Section 3**; lines **160-163; 166-170; 184-193**). We have also rewritten the section of the methods that describes our SVM classification in such a way that it will hopefully now be understandable to readers with no background in the methodology (lines **657-672**).

6. Lastly, we end up with, yet another set of genes associated with castes. Besides the GO terms, perhaps it would be good to see whether this set of 2000 genes found through machine learning actually match what is already known from previous caste gene expression studies who have looked closely at caste-specific gene expression in your study organism.

We have now expanded upon our discussion of the genes identified by the SVM approach (see response to Reviewer 2, Comment 5), as part of which we have compared this set of

genes to both a set of caste-biased genes and a set of vitellogenin-correlated genes from a previous gene expression study using *P. dominula* (Standage et al 2016; lines **497-501; 507-509**).

Minor comments

Introduction

7. I find the introduction well written overall, perhaps more examples of machine learning studies and how they are applied to transcriptomic/behavioral data would enable the readers to completely adhere to your methods.

While we lack space in the introduction to give a full overview of the kinds of studies that have used SVM classification, we have amended the manuscript to very briefly describe the methodology used by Chakravarty et al (2019), who we feel showcase a straightforward use for SVMs using easily-understood data (lines **123-124**).

8. L 122. "given their sensitivity" what do the authors mean there? Sensitive to changes in expression? How so?

For clarity, we have rephrased this to "given their ability to detect subtle changes in patterns of high-dimensional data" (lines **124-125**).

Methods

9. L. 426 – The filtering threshold seems a bit arbitrary, what does 20 counts represent in terms of cpm? Is it a threshold that is recommended for Support Vector machine classification?

While the threshold that we applied here is indeed partially arbitrary (as indeed are virtually any pre-filtering thresholds applied to gene count data), we feel that this threshold is statistically justified and additionally has the benefit of being rather conservative in the retention of genes. The DESeq2 user guide, for example, recommends a minimum of 10 reads/gene across all samples, which is genuinely arbitrary since this fails to take into account the respective sizes of comparison groups (Love et al 2020). Our threshold, by comparison, requires a minimum of 20 reads in at least one comparison group, meaning that we do not retain any gene whose expression is so low across all groups that it would not be possible to assess the presence of differential expression (which would unfairly bias against standard differential expression analysis methods such as DESeq2).

10. L. 453 – why using such stringent cut-off? Are there any differences in the number of DEGs when omitting this threshold and relying exclusively on pvalue?
11. L. 180 – Perhaps the low number of DEGs is also due to your cut off at LogFC 1.5 – how many more genes do you get when you include all genes with a significant transformed pvalue? Do they also belong to your list of 1992 genes from the SVM analysis?

We have amended the manuscript to address the effect of this fold change threshold (**General Changes, Section 2**; lines **224-231**; **289-291**).

12. L. 460 – WGCNA has an in-house filtering option that removed genes with too few values. Why did you decide to use your own filtering threshold? Or is this 10% threshold based on actual WGCNA analysis? Do the two approaches differ in the number of genes left after filtering? I recognize that WGCNA recommend a 10% threshold minimum, however the threshold used in a study should be based on experimental design, sequencing depth and sample counts.

As stated by the reviewer, the pre-filtering threshold that we selected is that recommended by the authors of the WGCNA package. We have updated the text to clarify that this was our reason for selecting this particular filtering threshold (lines **696-697**). We have also updated the text to give information as to how many additional genes were removed using this more stringent pre-filtering threshold (lines **697-698**).

The reviewer correctly points out that the WGCNA package authors recommend adjusting the pre-filtering threshold according to sequencing depth and sample counts. Compared to the microarray-derived dataset for which WGCNA was originally developed our data had a smaller sample size but a greater sequencing depth—on balance, we opted to use the default threshold recommended by WGCNA in order to maximise the comparability of our results to those of other papers using the recommended filtering threshold.

13. L 463 – Would it be possible to add some figures of the WGCNA methods? Such as soft-threshold power figure and the eigengene correlation figure to merge the modules?

We have added these two figures to the supplementary materials. **Figure S11** gives a set of soft-threshold figures that show the relationship between choice of soft threshold values and model fit/network connectivity. **Figure S12** gives a gene dendrogram with consensus modules shown.

14. Also, were the data transformed before being used for WGCNA analysis as recommended by the authors?

Data were subjected to variance stabilizing transformation (using DESeq2's built-in function) prior to analysis, as recommended by the package authors (Langfelder & Horvath 2014). We have updated the text to clarify this (lines **698-699**).

15. L. 469 – For the GO terms analysis, is the genome used in this study annotated? What about genes that do not have orthologues with D. melanogaster? That could lead to a bias in your results. How many genes were assigned orthologs? Doesn't NCBI provide quite a comprehensive annotation for every published genome? Why not using this?

Our motivation for using *D. melanogaster* orthologues rather than the GO data available for *P. dominula* was in fact to reduce the bias of our results. While the gene ontology data for *D. melanogaster* are highly detailed (at least relative to the vast majority of species), we speculated that those available for paper wasps were likely to be strongly biased towards social functions, since this species is a model for social complexity but is little studied with respect to other traits such as aging. As the reviewer points out, this approach may have introduced biases of its own if genes vary systematically in their likelihood of possessing orthologues in *D. melanogaster*, a possibility that we cannot rule out definitively. However, given that we found *D. melanogaster* orthologues for a fairly high proportion (62%) of genes, we considered the benefits of using GO terms from *D. melanogaster* to outweigh the possible downsides of this approach.

Results

16. L. 163 – is 25% a good value? Without background on the technique it is very difficult for the reader to make up its mind about this result (if this is even important).

We consider 25% a good value because it indicates a reliable separation between queens and workers: if workers rarely classify above 0.25, and queens rarely classify below 0.75, then the SVM placements of all control individuals are unambiguous (workers always classify closer to all other workers than to any queen, and vice versa). We have updated to the text to make this interpretation clearer to the reader (lines **180-182**).

17. L. 185 – You are basically testing only one method. If you would run the DEG analysis with a different software you might get a bigger data set, and perhaps those genes would not be

included in your final SVM dataset. To me it is hard to judge whether your SVM technic really captures almost all the information.

This is a fair criticism of the manuscript. We have only compared our SVM approach to a single alternative (DESeq2), which is one among many- others that we have used ourselves include edgeR and limma. Nonetheless, while these different programs do tend to produce somewhat divergent results to one another, their approaches are fundamentally very similar: differential expression is assessed on a gene-by-gene basis, with the power of the analysis boosted by fitting a means-variance curve that is then used to estimate the dispersion of each gene's expression as a function of its baseline expression. The nature of this type of approach is that it makes it very easy to assess and rank differential expression at the level of individual genes, but with little capacity to describe large-scale changes in gene expression: beyond the estimation of a means-variance curve (which itself is usually assumed not to vary between treatments), these programs do not employ any information sharing between genes. We thus feel that the general argument of our manuscript, namely that an SVM approach has an advantage over a standard differential expression approach in terms of predictive power when taking the entire transcriptome into account, is likely to apply regardless of the specific software employed.

18. L. 194 – is that a method section?

We are not sure what this comment is referring to, unless the reviewer means that they are unclear as to the meaning of the reference to Taylor et al (2020)—this paper is listed in the references section of the manuscript.

19. L. 200 – Were the nest used as a random factor in the DEG analysis?

Unfortunately, to our knowledge neither DESeq2 nor any of the other GLM-based differential expression programs (e.g. edgeR) currently support the use of random effects. While the degree of within-nest replication in this experiment is low (2-3 individuals per nest), we did attempt to assess the impact of nest effects using the duplicateCorrelation function from the Limma package (Ritchie et al 2015). Doing so, we found that the effect of nest identity upon individuals' gene expression profiles is very small (consensus within-nest correlation = 6.7%). This suggests to us that our inability to include a random effect in our models is unlikely to have been strongly impactful upon the results achieved.

20. L. 208 – The following paragraphs could be moved to the discussion, at least part of it sound very much like a discussion

While we do agree that this section could itself fit into the discussion, we feel that its current position provides useful context for the analyses that follow, which may be helpful in aiding readers to follow the argument of the paper. We would therefore argue that it is worth leaving this paragraph in its current position.

21. L 326. Once again, I do not understand what classifiers are, and what their variation really means, so the figure is quite obscure to me.

We have amended the results section and the caption to Figure 1 to make it clearer what we mean when we refer to classifiers (lines **166-170; 204-206**).

22. L. 275 – The WGCNA analysis. Were genes included in your three modules of interest also part of your 1922 genes from the SVM analysis? Is WGCNA a comprehensive approach to capture genes involved with the caste specific gene expression profiles and following perturbation?

We have added a supplementary table that gives the overlap of each GO module with the set of SVM-identified genes (**Table S6**), and we have confirmed in the text that each of the modules of interest does indeed overlap significantly with these (lines **352-354**). WGCNA does not, however, provide a means with which to predict the caste identity of individuals following queen removal, nor does any one of our WGCNA modules capture the full effects of queen loss.

Discussion

23. The authors found quite a large set of genes (around 20% of the total amount of genes in the genome) that were associated with the caste profiles using machine learning. We do not however understand how these genes are "associated", are they associated with one of the castes? With their behavior/phenotypic traits?

When we say that these genes are associated with caste, we mean that the genes contribute meaningfully to our ability to predict whether a given sample from our control data is a queen or a worker. To make this clearer, we have amended parts of the results and discussion (lines **185-188; 557-560**).

24. What about the WGCNA results? How do they fit with your hypothesis and conclusions? I cannot see a mention of them in the discussion

We have now incorporated the most interesting of our WGCNA results—the finding of a module that correlates with transcriptomic but not phenotypic caste identity and is enriched for genes associated with chromatin remodeling—into our results section (lines **484-492**). We feel that this result fits nicely with Reviewer 2's intriguing suggestion that post-removal changes reflect a form of transcriptomic priming.

<u>Reviewer #4 (Remarks to the Author):</u>

The manuscript "The molecular basis of socially-mediated phenotypic plasticity in a eusocial paper wasp" explores how brain gene expression shifts in adult wasps as they transition from a queenright (where all workers are subordinate) to a queenless (where workers compete for reproductive dominance) colony. The authors use a unique method to classify individuals on a one-dimensional linear scale ("queenness") based on behavioural (dominance index) and physiological (ovarian index) data. The results show that the 'queenness' index, as well as age can be used to explain the shift and variation in brain gene expression among queenless workers compared to queenright workers. Moreover, the authors highlight three modules of genes (and GO terms) that are co-expressed and correlate with queenness among workers in queenless colonies. The novelty and the broadness of scope are clearly outlined in the introduction and discussion. Moreover, by using Bayesian logistic regression model and SVM approach, the authors took complex behavioural, physiological, and genomic data, and simplified them to compare caste (queenness, Bayesian) and gene expression (SVM classifying module). The results were clearly presented, and conclusions drawn from those results were clear. Overall, the methods and the results can be built upon in future studies that explore how behavioural and physiological shifts may correspond to changes in gene expression - in a variety of animal systems, including, but not limited to, other species of social insects.

I only have a few minor comments that the authors may want to consider.

1. Line 59 & 376: Should this term be "bases" or "basis"?

Here we mean bases as the plural of basis, but we appreciate that this is a relatively uncommon plural form. To reduce the chances of confusion, we have replaced both instances of 'bases' with 'foundations' (lines **30; 61**).

2. Line 108: Consider removing "To do so," as the clause it is referring to is in the previous paragraph.

Thank you for pointing this out. This line has been changed as requested (line **109**).

3. Line 109: The methods make it clear that the whole brain transcriptome is being sequenced, but here "bran gene expression profiles" is used. It wasn't always clear that this was the whole transcriptome until line 112 (even though it's alluded to in line 87 as well). I think somehow this could be clarified (perhaps line 109 "assaying the transcriptome of the brain...").

As suggested, we have edited this sentence to directly refer to brain transcriptomes rather than gene expression profiles (line **110**).

4. Line 159: Similar to the point above "We trained an SVM using gene expression data..." It might be clearer if it said "whole transcriptome data" (to highlight the scope of the project).

Changed as suggested (line **175**).

5. Figure 1 tells an important part of the narrative, that overall brain gene expression shifted after queen removal. However, after reading about the "queenness" index, it seemed a bit odd that this was not a figure with the x-axis as "queenness" and the y-axis as the classifier estimate (as is shown in Fig 2).

We believe that for the intended purposes of **Figure 1**, maintaining a simple x-axis is a preferable approach for the following reasons:

- The concept of SVM classification is likely to be new to a significant proportion of readers, and we wish this first figure to serve as an introduction to the concept. In this sense, it is particularly important that this figure show clearly and unambiguously that our classifier separates queens and workers, for which purpose it is necessary to have these groups separated on the x-axis.
- One of the key results of the paper is that individuals from post-removal nests exhibit relatively consistent shifts in gene expression, and that these shifts do not differ greatly between individuals sampled at day 3 and at day 12 following queen removal. Again, having treatment group clearly delineated by the x-axis helps emphasise this result.

6. Figure 2 was a bit complicated. Perhaps A-C could be combined with Figure 1 instead?

- *Check colour codes in A-C. Blue now represents something different than in Fig 1. The colour codes also shifted in the Supplemental Figure.*
- *Y-axis is SVM Classifier Index (this took a minute to figure out from the caption)*
- *Is there a reason why Age v Queenness figure is not shown? Perhaps as an inset? The relationship is alluded to in the discussion as well (lines 308-9).*
- *D-F: Why are there colours used to name the modules? It might be easier to interpret if they were just numbers or letters. Also - consider placing the column titles on top, and labeling the first column (with the numbers:colours ID) as "Module". I didn't notice it as a title on the left.*
- *G-H: please define BP, CC, and MF in the figure caption.*

Having discussed the reviewer's helpful comments regarding the figures in the manuscript, we have made a large number of alterations to the layout of these figures, as follows:

- All figures that compare treatment groups (**Figures 1 and 2 and supplementary figures**) now use a standardised colour coding for each group
- The Y-axis text for **Figure 2** has been amended to make it clearer that the measure shown is the SVM classification
- Module-trait correlation figures have been amended as requested: modules are now referred to only as numbers (not colour codes), trait names are listed at the top of the figure instead of the bottom, and the order of the modules have been reversed to accommodate a top-to-bottom reading (**Figures S6-S8**)
- Gene ontology plots now properly define the three GO domains in their legends (**Figures S2, S9-S10**)

Additionally, the comments of the reviewers have brought to our attention the overall difficulty of reading for **Figure 2**, which perhaps attempts to convey too much information in a small space. To emphasise the most important result given by this figure (the relationship between SVM classification and phenotypic caste/age), we have moved the module-trait correlation and module gene ontology figures to the supplementary materials.

7. Lines 275-292: The order in which these modules are presented is awkward - particularly since 11 and 8 are highlighted in Figure 2, and 2 is discussed in the middle of the paragraph.

We have re-ordered this paragraph to logically follow the ordering of modules in the figures (lines **356-378**). Please note that we have also amended this paragraph slightly to reflect changes in the GO assignments for modules 8 and 11, which we discovered in the course of this revision (lines **363; 369**)—these discrepancies were the results of our GO analysis for the modules previously using an inappropriate background gene universe which failed to take into account the additional filtering applied prior to WGCNA analysis.

References

Bengston, S.E., Dahan, R.A., Donaldson, Z., Phelps, S.M., Van Oers, K., Sih, A., & Bell, A.M., 2018. Genomic tools for behavioural ecologists to understand repeatable individual differences in behaviour. Nature Ecology & Evolution, 2(6), 944-955.

Chalfin, L., Dayan, M., Levy, D.R., Austad, S.N., Miller, R.A., Iraqi, F.A., Dulac, C. and Kimchi, T., 2014. Mapping ecologically relevant social behaviours by gene knockout in wild mice. Nature communications, 5(1), pp.1-10.

Gacias, M., Gaspari, S., Santos, P.M.G., Tamburini, S., Andrade, M., Zhang, F., Shen, N., Tolstikov, V., Kiebish, M.A., Dupree, J.L. and Zachariou, V., 2016. Microbiota-driven transcriptional changes in prefrontal cortex override genetic differences in social behavior. elife, 5, p.e13442.

Langfelder, P. and Horvath, S., 2014. Tutorial for the WGCNA package for R. https://horvath.genetics.ucla.edu/html/CoexpressionNetwork/Rpackages/WGCNA/Tutorials

Love, M.I, Anders, S. and Huber, W., 2020. Analyzing RNA-seq data with DESeq2. http://bioconductor.org/packages/devel/bioc/vignettes/DESeq2/inst/doc/DESeq2.html

Makhijani, R.K., Raut, S.A. and Purohit, H.J., 2018. Fold change based approach for identification of significant network markers in breast, lung and prostate cancer. IET systems biology, 12(5), pp.213-218.

Staudt, M.R., Salit, J., Kaner, R.J., Hollmann, C. and Crystal, R.G., 2018. Altered lung biology of healthy never smokers following acute inhalation of E-cigarettes. Respiratory research, 19(1), p.78.

Valcu, C.M., and Kempenaers, B., 2015. Proteomics in behavioral ecology. Behavioral Ecology, 26(1), 1-15.

Reviewer #1 (Remarks to the Author):

This revised manuscript provides creative statistical approaches to an interesting data set, but after revision the story that the authors are supporting is somewhat difficult for me to articulate clearly. The two main questions I took away from the study are 1) how is trait variation that is influenced by queen removal reflected or caused at the molecular level (via brain gene expression), and 2) how is brain gene expression linked to 2 trait measurements in the focal individuals? The authors use queens and workers to train a model (SVM) to then score transcriptomes in the focal individuals. They argue that SVM is superior to traditional statistical approaches but that it is difficult to link genotype to phenotype because many gene expression differences may not be relevant to the traits. This is indeed a challenging problem for geneticists (one paper that came is https://doi.org/10.1093/gbe/evr004). Unfortunately, it is challenging to see what is learned about 1) the molecular basis of trait variation of interest or 2) why the SVM approach provides particularly useful insights that would support its adoption in other, similar studies. Sometimes the messaging seems a bit contradictory - it is argued that most gene expression changes are unlinked to trait variation but also that the SVM approach is providing useful information in generating a larger list of such genes, which may in turn help to understand either the molecular basis of trait variation or to assign individuals to trait categories based on molecular information.

Reviewer #2 (Remarks to the Author):

The authors have done an excellent job addressing all of the points raised in my review and I have no further concerns.

Reviewer #3 (Remarks to the Author):

This is the second time that I review this manuscript. I applaud the authors for a very detailed and thorough revisions and for their nice replies.

The authors have addressed all of the issues that I raised in my previous review. They have extensively rewritten the manuscript, made understanding the methods much easier and added supplementary materials.

Overall, I am happy with the revisions and I would recommend to the editor that the manuscript be accepted.

Reviewer #4 (Remarks to the Author):

The revised manuscript "The molecular basis of socially-mediated phenotypic plasticity in a eusocial paper wasp" provides a lot of additional detail to help clarify the methods and analyses. As mentioned in my initial review, I'm really impressed and excited about this study, particularly that it takes complex data and simplifies them into single axes for clear comparisons across treatment groups, which lead to clear conclusions.

The only minor item to note is to do with the color scheme in the figures and boxes. Figure 1 & 2 match, but the colors in Box 1 (queen and control workers) are switched from the black and gold used in the figures.

Response to referees: The molecular basis of socially-mediated phenotypic plasticity in a eusocial paper wasp (Ref: NCOMMS-20-28189B)

We would like to once again thank the four anonymous reviewers for taking time to comment on our manuscript. We detail our responses to the modifications requested by reviewers 1 and 4 below. Line numbers refer to the copy of the revised manuscript with tracked changes.

(*reviewer comments are in italics*; author responses are in roman case)

Reviewer #1:

This revised manuscript provides creative statistical approaches to an interesting data set, but after revision the story that the authors are supporting is somewhat difficult for me to articulate clearly. The two main questions I took away from the study are 1) how is trait variation that is influenced by queen removal reflected or caused at the molecular level (via brain gene expression), and 2) how is brain gene expression linked to 2 trait measurements in the focal individuals? The authors use queens and workers to train a model (SVM) to then score transcriptomes in the focal individuals. They argue that SVM is superior to traditional statistical approaches but that it is difficult to link genotype to phenotype because many gene expression differences may not be relevant to the traits. This is indeed a challenging problem for geneticists (one paper that came is https://doi.org/10.1093/gbe/evr004). Unfortunately, it is challenging to see what is learned about 1) the molecular basis of trait variation of interest or 2) why the SVM approach provides particularly useful insights that would support its adoption in other, similar studies. Sometimes the messaging seems a bit contradictory - it is argued that most gene expression changes are unlinked to trait variation but also that the SVM approach is providing useful information in generating a larger list of such genes, which may in turn help to understand either the molecular basis of trait variation or to assign individuals to trait categories based on molecular information.

We thank the reviewer for this comment. In the manuscript, we provide two separate arguments in favour of the SVM approach: first, that this approach provides a less subjective means of assessing differential expression between two well-defined treatment groups than do standard approaches; and second, that the classifier generated using this approach can be productively applied to individuals outside these groups to reveal aspects of gene expression that would otherwise not be apparent. Although these two arguments are not contradictory, we do appreciate that their presentation within the manuscript may lead to a degree of confusion, because the classifications generated by applying our SVM model to the test data set include an effect that appears to be only partially structured by the phenotypic correlates of caste expression. To address this issue with the presentation, we have amended the first and last paragraphs of the discussion (lines 385-387; 533-536) in order to emphasise the fact that our results support two related but distinct applications of SVMs: identification of variables (e.g. genes) that meaningfully predict a response of interest (e.g. caste phenotype); and identification of broad-scale shifts in variable profiles (e.g. transcriptomes) that follow a change in the response. We hope that these amendments will make it clearer that these two applications are not in fact contradictory.

Reviewer #4:

The revised manuscript "The molecular basis of socially-mediated phenotypic plasticity in a eusocial paper wasp" provides a lot of additional detail to help clarify the methods and analyses. As mentioned in my initial review, I'm really impressed and excited about this study, particularly that it takes complex data and simplifies them into single axes for clear comparisons across treatment groups, which lead to clear conclusions.

The only minor item to note is to do with the color scheme in the figures and boxes. Figure 1 & 2 match, but the colors in Box 1 (queen and control workers) are switched from the black and gold used in the figures.

We thank the reviewer for pointing out this discrepancy in the figures' color schemes. We have edited Figures 2 and 3 (formerly 1 and 2) to match the color scheme for queens and workers first set out in Figure 1 (formerly Box 1).
